# Four New Pterosins from *Pteris cretica* and Their Cytotoxic Activities

**DOI:** 10.3390/molecules24152767

**Published:** 2019-07-30

**Authors:** Jian Lu, Caiying Peng, Shuang Cheng, Jianqun Liu, Qinge Ma, Jicheng Shu

**Affiliations:** Key Laboratory of Modern Preparation of Traditional Chinese Medicines, Ministry of Education, Jiangxi University of Traditional Chinese Medicine, Nanchang 330004, China

**Keywords:** *Pteris cretica* Linn., pterosins, cytotoxic activity

## Abstract

Phytochemical investigation of the aerial parts of *Pteris cretica* led to the isolation and elucidation of nine pterosins, including four new pterosins, creticolacton A (**1**), 13-hydroxy-2(*R*),3(*R*)-pterosin L (**2**), creticoside A (**3**), and spelosin 3-*O*-β-d-glucopyranoside (**4**), together with five known pterosins **5**–**9**. Their structures were identified mainly on the basis of 1D and 2D NMR spectral data, ESI-MS and literature comparisons. Compounds **1** and **3** were new type of petrosins with a six membered ring between C-14 and C-15. The new compounds were tested in vitro for their cytotoxic activities against four human tumor cell lines (SH-SY5Y, SGC-7901, HCT-116, Lovo). Results showed that compounds **1** and **2** exhibited cytotoxic activity against HCT-116 cells with IC_50_ value of 22.4 μM and 15.8 μM, respectively.

## 1. Introduction

The genus *Pteris* (Pteridaceae) comprises about 300 species, which are mainly distributed over the tropical and temperate zones throughout the world. There are about 66 species of the genus in China, especially in South and Southwest of China [1]. *Pteris* is commonly known as “Jue-Cai” in Chinese. Most species of this genus such as *Pteris multifida*, *Pteris cretica*, and *Pteris nervosa* are used as traditional Chinese medicines to clear heat, remove dampness and cool blood in medical practice [2]. In recent years, the phytochemical investigation of this genus has resulted in the isolation and identification of sesquiterpenoids (known as pterosins), diterpenes, flavonoids and various quinic acids [3,4,5]. Among of above secondary metabolites, pterosins, illudane-type sesquiterpenoids are most significant characteristic constituents of *Pteris*. Moreover, pterosins are used as the chemical markers for Pteridaceae [6]. Pterosins can be classified into three categories according to the number of skeletal carbon, such as 13 carbon, 14 carbon and 15 carbon pterosin derivatives [6,7]. Pharmacological investigations showed that pterosins had many bioactivities, such as antitumor, anti-inflammatory, anti-diabetes and anti-tuberculosis properties [8,9,10,11]. However, most studies on pterosins from this genus focused on *Pteris multifida* [7]. Other species of the *Pteris* genera probably possess pharmacological activities and bioactive secondary metabolites that are similar to *Pteris multifida*. As a part of our ongoing research aimed at finding potentially bioactive components from medicinal plants of the genus *Pteris* [12,13,14], we have carried out a detailed phytochemical investigation of the aerial parts of *Pteris cretica* Linn. (*P. cretica*). Herein described are the isolation and structural elucidation of nine pterosins, including four pterosins (compounds **1**, **2**, **5** and **6**), along with five pterosides (compounds **3**, **4** and **7**–**9**).

## 2. Results and Discussion

**Figure 1:**
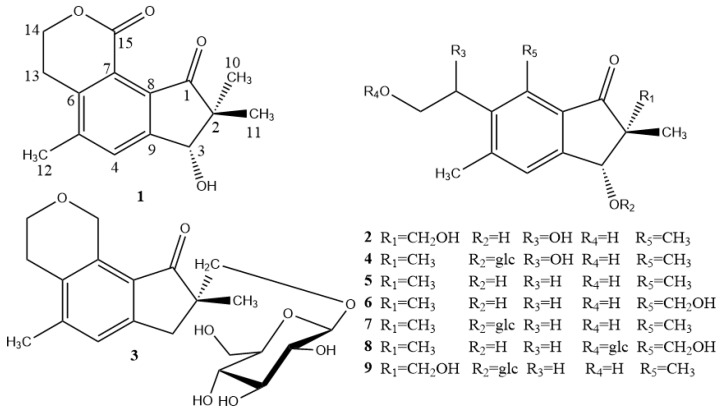
Structures of compounds **1**–**9**.

The 70% ethanol extract of *P. cretica* was extracted with petroleum ether, dichloromethane, EtOAc and *n*-BuOH, separately. The *n*-BuOH extraction was repeatedly subjected to thin-layer, normal-phase column and reversed phase semi-preparative column chromatography to afford four new pterosins **1**–**4**, together with five known pterosins, namely pterosin D (**5**) [15], (3*R*)-pterosin W (**6**) [8], (3*R*)-pterosin D 3-*O*-β-d-glucopyranoside (**7**) [16], 3(*R*)-pteroside W (**8**) [17], 2*R*,3*R*-pterosin L 3-*O*-β-d-glucopyranoside (**9**) [18]. The structures of compounds **1**–**9** are illustrated in Figure 1.

**Figure 2:**
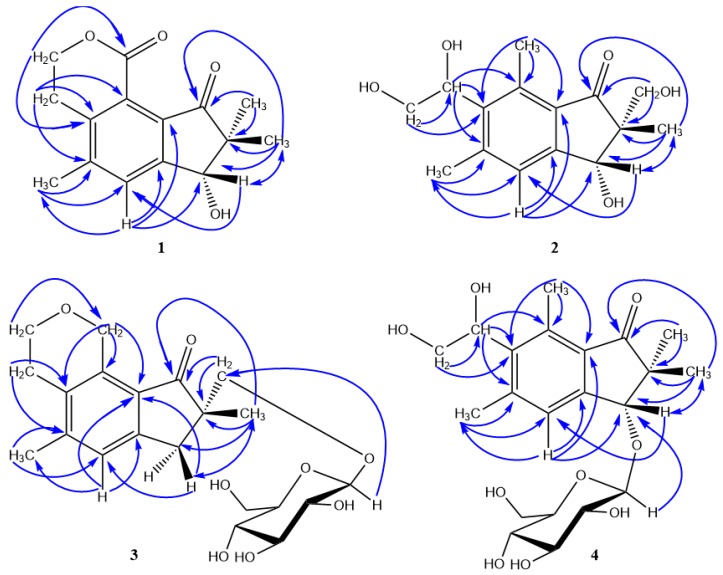
The key HMBC (→) and NOESY (↔) of compounds **1–4**.

Compound **1** was isolated as colorless amorphous powder (MeOH) and showed a molecular formula of C_15_H_16_O_4_ with eight degrees of unsaturation, as determined by a HRESI-MS peak at *m/z* 261.1127 [M + H]^+^ (calcd for C_15_H_17_O_4_, 261.1127) (See Supplementary Materials). The UV spectrum exhibited the characteristic absorptions of pterosin-type sesquiterpenes at 215 (4.03), 258 (3.71), 310 (2.97) nm [15]. In the ^1^H-NMR spectrum (Table 1), three singlet methyl signals, including two geminal dimethyl groups located at C-2 (*δ*_H_ 1.10 and 1.25, each 3H) and one aromatic methyl group (*δ*_H_ 2.49, 3H), a carbinyl proton at C-3 (*δ*_H_ 4.80, s), an aromatic proton (*δ*_H_ 7.80, s), and two methine groups [*δ*_H_ 3.06 (2H, m, H-13) and 4.55 (1H, m, H-14a), 4.49 (1H, m, H-14b)] were observed. The ^13^C-NMR displayed 15 carbon resonances (Table 1), including three methyls at *δ*_C_ 20.0 (C-10), *δ*c 20.3 (C-12) and *δ*c 22.6 (C-11), two methylenes at *δ*c 27.0 (C-13) and *δ*c 67.9 (C-14), two methines at *δ*c 77.1 (C-3) and *δ*c 132.4 (C-4), and eight quaternary carbons. Comparison of the NMR data of **1** with those of pterosin D (**5**) [15], which was isolated at the same time, revealed that they shared a similar structure. The differences between them were that one ester carbonyl group (*δ*c 163.5) replacing the methyl group (C-15) of pterosin D was observed in **1**. The proton and carbon chemical shifts of C-14 appeared at the low field region due to the deshielding influence of the oxygenatom suggested **1** possessed a lactone link between positions C-14 and C-15. This was further confirmed by the HMBC spectrum (Figure 2) that showed the correlations from H-14 (*δ*_H_ 4.49, 4.55) to C-15 (*δ*_C_ 163.5). The absolute configuration of **1** was also determined as 3*R* by the CD spectrum which exhibited a positive cotton effect at 326 nm [19]. Based on these results, compound **1** was identified as creticolactone A, which represents the new type of petrosin possessing a six membered lactone ring between positions C-14 and C-15.

Compound **2** was isolated as colorless amorphous powder (MeOH) and showed a molecular formula of C_15_H_20_O_5_ with six degrees of unsaturation as determined by the HRESI-MS peak at *m/z* 315.1001 [M + Cl]^−^ (calcd for C_15_H_20_O_5_Cl, 315.0999) (See Supplementary Materials). The UV spectrum exhibited the characteristic absorptions of pterosin-type sesquiterpenes at 222 (4.19), 265 (3.82), 305 (2.78) [15]. In the ^1^H-NMR spectrum (Table 1), three singlet methyl signals, including one geminal dimethyl group located at C-2 (*δ*_H_ 1.15) and two aromatic methyl groups (*δ*_H_ 2.58 and 2.76), a carbinyl proton at C-3 (*δ*_H_ 4.84) and an aromatic proton (*δ*_H_ 7.35) were observed.

The ^13^C-NMR displayed 15 carbon resonances, including three methyls at *δ*c 15.2 (C-15), *δ*c 19.2 (C-11) and δc 22.4 (C-12), two oxygenated methylenes at *δ*c 65.6 (C-14) and *δ*c 67.1 (C-10), three methines at *δ*c 72.7 (C-13), 77.4 (C-3) and *δ*c 126.9 (C-4), and eight quaternary carbons. The ^1^H-NMR and ^13^C-NMR spectra of compound **2** showed similar features to those of 2(*R*),3(*R*)-pterosin L [15]. The difference between the ^1^H-NMR of **2** and that of 2(*R*),3(*R*)-pterosin L was that 2(*R*),3(*R*)-pterosin L exhibited an A_2_X_2_ coupled system (2H × 2, t, *J* = 8.0 Hz), but **2** showed characteristic ABX signals [*δ*_H_ 5.32 (1H, dd, *J* = 4.9, 8.3 Hz, H-13), 3.93 (1H, dd, *J* = 8.3, 11.4 Hz, H-14a), 3.64 (1H, dd, *J* = 4.9, 11.4 Hz, H-14b)]. This suggested a 1,2-glycol was located at C-6 of **2**. This was also confirmed by the carbon chemical shifts of C-13 (*δ*_C_ 72.7) appearing in the low field region due to the deshielding influence of the oxygen atom. Furthermore, in HMBC spectrum (Figure 2), the methine proton at *δ*_H_ 5.32 (H-13) showed cross-peaks with C-5, C-6 and C-7, which further indicated that the attached hydroxyl group could be assigned to C-13. The *cis*-configuration of the methyl at C-2 and the H at C-3 in **2** was confirmed by NOESY correlations between H-3 and H-11. The absolute configuration of **2** was determined by its CD spectrum, which exhibited a positive Cotton effect at 329 nm in MeOH, indicating that the oxygenated group at C-3 existed in a pseudoaxial conformation irrespective of the configuration at C-2 [14,19]. Based on these results, compound **2** was assigned as 13-hydroxyl-2(*R*),3(*R*)-pterosin L.

Compound **3** was isolated as a colorless amorphous powder (MeOH) and showed a molecular formula of C_21_H_28_O_8_ with eight degrees of unsaturation as determined by a HRESI-MS peak at *m/z* 431.1672 [M + Na]^+^ (calcd. for C_21_H_28_O_8_Na, 431.1682) (See Supplementary Materials). The UV spectrum exhibited the characteristic absorptions of pterosin-type sesquiterpenes at 228 (4.28), 270 (3.99), 310 (3.27) nm [15]. The ^1^H-NMR spectrum (Table 2) showed signals of one singlet methyl (*δ*_H_ 1.14, H-11), one aromatic methyl (*δ*_H_ 2.35, H-12), a methylene group [2.80 (d, *J* = 17.0 Hz, H-3a), 3.50 (d, *J* = 17.0 Hz, H-3b)], an aromatic proton [*δ*_H_ 7.22 (s, H-4)], an ethanol grouping located at C-6 [*δ*_H_ 2.74 (2H, t, *J* = 5.5 Hz, H-13), 3.98 (2H, m, H-14)], and two oxygenated methylene groups [*δ*_H_ 4.10 (1H, d, *J* = 9.4 Hz, H-10a), 3.48 (1H, d, *J* = 9.4 Hz, H-10b), 5.08 (2H, d, *J* = 4.8 Hz, H-15)]. Furthermore, the presence of a β-configuration glucose moiety was evident from the signals assignable to one anomeric proton (*δ*_H_ 4.20, d, *J* = 7.9 Hz), two oxygenated methine protons [*δ*_H_ 3.78 (dd, *J* = 1.6, 11.8 Hz) and 3.61 (dd, *J* = 5.7, 11.8 Hz)], and four overlapping protons (*δ*_H_ 3.03–3.28) [20]. This was further confirmed by GC-MS analysis after acid hydrolysis [21]. The connectivity of the glucose part was identified by HMBC correlations between the H-glc-1 (*δ*_H_ 4.20) and the C-10 (*δ*_C_ 74.86). 

Compound **3** exhibited ^1^H- and ^13^C-NMR data closely resembling to those of rhedynoside A [22]. The difference between NMR spectroscopic data of **3** (Table 2) and those of rhedynoside A was **3** possesses an ether link. The presence of an ether link was assigned to be between positions C-15 and C-14 as their proton and carbon chemical shifts appeared at the low field region due to the deshielding influence of the oxygen atom. This was further confirmed by the HMBC spectrum that showed the correlations from H-15 (*δ*_H_ 5.08) to C-14 (*δ*_C_ 65.6) and from H-14 (*δ*_H_ 3.98) to C-15 (*δ*_C_ 67.5). The absolute configuration of **3** was also determined as 2*S* by the CD spectrum which exhibited vibronic *n*–π* transition which concurs with C-2 having 2*S* configuration [22]. Based on these results, compound **3** was assigned as creticoside A.

Compound **4** was isolated as colorless amorphous powder (MeOH) and showed a molecular formula of C_21_H_30_O_9_ with seven degrees of unsaturation as determined by a HRESI-MS peak at *m/z* 461.1581 [M + Cl]^−^ (calcd for C_21_H_30_O_9_Cl, 461.1578) and 471.1874 [M + COOH]^−^ (calcd for C_22_H_31_O_12_, 471.1866) (See Supplementary Materials). The UV spectrum exhibited the characteristic absorptions of pterosin-type sesquiterpenes at 219 (4.16), 265 (3.80), 303 (2.88) nm [15]. In the ^1^H-NMR spectrum (Table 2), four singlet methyl signals, including two geminal dimethyl groups located at C-2 (*δ*_H_ 1.08 and 1.28, each 3H) and two aromatic methyl groups (*δ*_H_ 2.57 and 2.74, each 3H), a carbinyl proton at C-3 (*δ*_H_ 4.84, s), an aromatic proton (*δ*_H_ 7.52, s), and a set of ABX signals [*δ*_H_ 5.32 (dd, *J* = 4.9, 8.4 Hz), 3.92 (dd, *J* = 8.4, 11.5 Hz), 3.63 (dd, *J* = 4.9, 11.5 Hz)] assignable to a 1,2-glycol located at C-6 were observed. Compound **4** exhibited ^1^H and ^13^C-NMR data closely resembling to those of spelosin [15]. Comparison of the UV and NMR spectroscopic data of **4** (Table 2) with those of spelosin showed several differences, the main one being the presence of an additional hexose sugar moiety having a signal for an anomeric-H at *δ*_H_ 4.57 (d, *J* = 7.8 Hz) and remaining sugar proton signals at *δ*_H_ 3.27–3.85. The ^13^C-NMR spectrum of **4** contained an anomeric carbon signal of a hexose moiety at *δ*_H_ 105.8 and signals for remaining five sugar carbons at *δ*_H_ 62.8–78.2, which were in good agreement with those reported for glucoside compounds [22]. Furthermore, the coupling constant (*J* = 7.8 Hz) is consistent with a *trans* ^3^*J*_H-H_, showing that the glucose moiety is in a β-configuration [20]. This was further confirmed by GC-MS analysis after acid hydrolysis [21]. The connectivity of glucose part was identified by HMBC correlations between the H-1″ (*δ*_H_ 4.57) and the C-3 (*δ*_C_ 86.0) (Figure 2). The absolute configuration of **4** was also determined as 3*R* by the CD spectrum which exhibited a positive Cotton effect at 329 nm [19]. Based on these results, compound **4** was assigned as spelosin 3-*O*-β-d-glucopyranoside.

Compounds **1**–**4** were evaluated for cytotoxic activities against four human cell lines (SH-SY5Y (neuroblastoma cell line), SGC-7901 (gastric cancer cell line), HCT-116 (colon cancer cell line) and Lovo (colorectal cancer cell line). Compounds **1**–**4** were inactive (IC_50_ > 100 μM) to SH-SY5Y, SGC-7901, and HCT-116 cell lines. Compounds **1** and **2** exhibited cytotoxic activity against HCT-116 cells with IC_50_ value of 22.4 μM and 15.8 μM, respectively.

## 3. Experimental Section

### 3.1. General Information

1D and 2D-NMR spectra were obtained on an AV-600 spectrometer (Bruker, Rheinstetten, Germany) with TMS as internal reference, and methanol-*d*_4_ as solvent. Electrospray ionisation (ESI) mass spectra were acquired in the positive ion mode on a LCQ DECAXP instrument (Thermo Finnigan, San Jose, CA, USA) equipped with an ion trap mass analyzer. HRESI-MS were obtained in the positive ion mode on a Waters UPLC Premier Q-TOF system (Waters Inc., Milford, MA, USA). CD spectra were obtained on an Olis DSM 1000 spectrometer (Olis Inc., Augusta, GA, USA). Optical rotations were acquired on Shenguang SGW-1 digital polarimeter (Jingke, Shanghai, China). GC-MS were obtained on a Thermo Finnigan Trace DSQ (TR-5MS column: 60 m × 0.25 mm × 2.5 μm) (ThermoFinnigan, Bremen, Germany). TLC plates were HSGF254 SiO_2_ from Yantai Jiangyou Silica Gel Development Co., Ltd. (Yantai, China). Column chromatography (CC) silica gel (SiO_2_; 200–300 mesh; Qingdao Haiyang Chemical Co., Ltd., Qingdao, China), Sephadex LH-20 (GE-Healthcare Bio-Sciences AB, Uppsala, Sweden), ODS (Grace C18, Grace Davison Discovery Sciences, Columbia, MD, USA) were employed as packing materials, semi-preparative HPLC (Grace Prevail C18 column, 5 μm, 10.0 mm I.D × 250 mm, Grace Davison Discovery Sciences, Columbia, MD, USA). All other chemicals were of analytical reagent grade.

### 3.2. Plant Materials

The aerial parts of *P. cretica* were collected in Jiangxi China, and identified by Prof. Xiaomei Fu of Jiangxi University of Traditional Chinese Medicine. A voucher specimen (NO. 20161007) was deposited at the Key Laboratory of Modern Preparation of TCM, Jiangxi University of Traditional Chinese Medicine, China.

### 3.3. Extraction and Isolation

The aerial parts of *P. cretica* (5 kg) were extracted with 70% (*v/v*) aqueous ethanol (about 60 L). The 70% EtOH extract was concentrated under reduced pressure to give a residue (803 g), which was then extracted with petroleum ether, dichloromethane, EtOAc and *n*-BuOH, respectively. The above extracts were concentrated to yield 81 g of petroleum ether fraction, 108 g of dichloromethane fraction, 154 g of EtOAc fraction and 132 g of *n*-BuOH fraction. The UV analysis of the above fractions monitoring the characteristic absorption bands in the 304 to 205 nm region of pterosin derivatives [15,16], showed that the *n*-BuOH extract contained more pterosins in comparison with other extracts. Therefore, the *n*-BuOH extract was subjected to further purification. Part of the dried *n*-BuOH extract (about 130 g) was subjected to silica gel column chromatography with gradient mixtures of CH_2_Cl_2_/ MeOH (from 10:1 to 1:10). Each 200 mL fraction was collected and evaporated, and some fractions was combined after analytical TLC inspection (CHCl_3_/MeOH 10:1 to 3:1). Finally, 33 fractions (*Fr.*1–33) were obtained. *Fr.*8 (1.7 g) was further subjected to silica gel CC (CH_2_Cl_2_/MeOH 10:1, 8:1, 4:1). Each 50 mL of eluent was collected and combined after analytical TLC inspection (CHCl_3_/MeOH 8:1 to 5:1). 12 fractions (*Fr.*8.1–12) were obtained. *Fr.*8.4 (256 mg) was further subjected to Sephadex LH-20 column chromatography with MeOH. 15 fractions (*Fr.*8.4.1–15) were obtained. *Fr.*8.4.6 (57 mg) was further purified by semi-preparative HPLC (65% MeOH/H_2_O, 3.0 mL/min) to yield **1** (7.2 mg, t_R_18.2 min), **5** (9.7 mg, t_R_14.5 min), **6** (11.1 mg, t_R_10.5 min). *Fr.*12 (2.2 g) was further subjected to silica gel CC (CH_2_Cl_2_/MeOH 9:1, 7:1, 3:1). Twenty-three fractions (*Fr.*12.1–23) were obtained. *Fr.*12.9 (195 mg) was further subjected to Sephadex LH-20 column chromatography with MeOH. Eighteen fractions (*Fr.*12.9.1–18) were obtained. *Fr.*12.9.5 (24 mg) was further purified by semi-preparative HPLC (59% MeOH/H_2_O, 3.0 mL/min) to yield **2** (6.8 mg, t_R_18.3 min). *Fr.*12.9.16 (21 mg) was further purified by semi-preparative HPLC (55% MeOH/H_2_O, 3.0 mL/min) to yield **3** (8.1 mg, t_R_15.6 min). *Fr.*18 (1.0 g) was further subjected to silica gel CC (CH_2_Cl_2_/MeOH 9:1, 7:1, 3:1). Twenty fractions (*Fr.*18.1–20) were obtained. *Fr.*18.6 (195 mg) was further subjected to Sephadex LH-20 column chromatography with MeOH. Eleven fractions (*Fr.*18.6.1–11) were obtained. *Fr.*18.6.5 (19 mg) was further purified by semi-preparative HPLC (50% MeOH/H_2_O, 3.0 mL/min) to yield **4** (7.3 mg, t_R_17.6 min). *Fr.*18.6.9 (25 mg) was further purified by semi-preparative HPLC (50% MeOH/H_2_O, 3.0 mL/min) to yield **7** (10.0 mg, t_R_15.5 min). *Fr.*22 (897 mg) was further subjected to Sephadex LH-20 column chromatography with MeOH. Sixteen fractions (*Fr.*22.1–16) were obtained. *Fr.*22.7 (68 mg) was further purified by semi-preparative HPLC (45% MeOH/H_2_O, 3.0 mL/min) to yield **8** (14.3 mg, t_R_15.3 min). *Fr.*22.11 (35 mg) was further purified by semi-preparative HPLC (45% MeOH/H_2_O, 3.0 mL/min) to yield **9** (15.0 mg, t_R_19.5 min).

### 3.4. Spectral Data

*Creticolactone A* (**1**): Colorless amorphous powder (MeOH). ${\left[\alpha \right]}_{D}^{25}$ = +51.4° (c =0.032, MeOH). UV (MeOH) λ_max_ (logε) 215 (4.03), 258 (3.71), 310 (2.97) nm; HRESI-MS at *m/z* 261.1127 [M + H]^+^ (calcd for C_15_H_17_O_4_, 261.1127); ^1^H-NMR and ^13^C-NMR see Table 1.

*13-Hydroxy-2(R),3(R)-pterosin L* (**2**): Colorless amorphous powder (MeOH). ${\left[\alpha \right]}_{D}^{25}$ = +30.0° (c =0.032, MeOH). UV (MeOH) λ_max_ (logε) 222 (4.19), 265 (3.82), 305 (2.78) nm; HRESI-MS at *m/z* 315.1001 [M + Cl]^−^ (calcd for C_15_H_20_O_5_Cl, 315.0999); ^1^H-NMR and ^13^C-NMR see Table 1.

*Creticoside A* (**3**): Colorless amorphous powder (MeOH). ${\left[\alpha \right]}_{D}^{25}$ = −32.0° (c =0.032, MeOH). UV (MeOH) λ_max_ (logε) 228 (4.28), 270 (3.99), 310 (3.27) nm; HRESI-MS at *m/z* 431.1672 [M + Na]^+^ (calcd for C_21_H_28_O_8_Na, 431.1682); ^1^H-NMR and ^13^C-NMR see Table 2.

*Spelosin 3-O-*β*-*d*-glucopyranoside* (**4**): Colorless amorphous powder (MeOH). ${\left[\alpha \right]}_{D}^{25}$ = −17.8° (c =0.032, MeOH). UV (MeOH) λ_max_ (logε) 219 (4.16), 265 (3.80), 303 (2.88) nm; HRESI-MS at *m/z* 461.1581 [M + Cl]^−^ (calcd for C_21_H_30_O_9_Cl, 461.1578); ^1^H-NMR and ^13^C-NMR see Table 2.

### 3.5. Cytotoxic Activity

The cytotoxicity of the isolated compounds (the purities of compounds **1**–**4** were 95.5%, 93.0%, 92.5% and 95.6%, respectively) against four human tumor cell lines (SH-SY5Y, SGC-7901, HCT-116, Lovo) was evaluated using the MTT assay performed according to the method described by Liu [23].

### 3.6. Acid Hydrolysis of ***3***–***4***

The procedure of the absolute configuration determination of glucose (compounds **3** and **4**) was as previously reported [21].

## 4. Conclusions

This study describes the successful isolation and identification of nine pterosin-type sesquiterpenoids from the aerial part of *P. cretica*, including four new pterosins (compounds **1**–**4**). Their chemical structures were elucidated by 1D- and 2D-NMR spectroscopic analysis. creticolactone A (**1**) and creticoside A (**3**) belong to a new type of pterosin with a six membered ring between positions C-14 and C-15. To our best knowledge, this is the first report on the occurrence of this new class of pterosins from the genus *Pteris*, which implies that they might be chemotaxonomic markers for *P. cretica*. Furthermore, all of the new isolated compounds **1**–**4** were evaluated for cytotoxic activity. Compounds **1** and **2** exhibited cytotoxic activity against HCT-116 cells with IC_50_ values of 22.4 μM and 15.8 μM.

## Figures and Tables

**Table 1:** The NMR spectroscopic data of compounds **1** and **2** (MeOH-*d*_4_, ^1^H-NMR 600 MHz, ^13^C-NMR 150 MHz).

Position	1		2	
	*δ*_H_ (*J* in Hz)	*δ*_C_	*δ*_H_ (*J* in Hz)	*δ*_C_
1	-	206.1	-	209.7
2	-	53.4	-	57.0
3	4.80 (s)	77.1	4.84 (s)	77.4
4	7.80 (s)	132.4	7.35 (s)	126.9
5	-	144.4	-	146.6
6	-	144.1	-	140.4
7	-	123.9	-	138.2
8	-	134.0	-	133.1
9	-	155.1	-	155.6
10	1.10 (s)	20.0	3.73 (d, *J* = 11.0)	67.1
			3.68 (d, *J* = 11.0)	
11	1.25 (s)	22.6	1.15 (s)	19.2
12	2.49 (s)	20.3	2.58 (s)	22.4
13	3.06 (m)	27.0	5.32 (dd, *J* = 4.9, 8.3)	72.7
14	4.49 (m)	67.9	3.93 (dd, *J* = 8.3, 11.4)	65.6
	4.55 (m)		3.64 (dd, *J* = 4.9, 11.4)	
15	-	163.8	2.76 (s)	15.2

**Table 2:** The NMR spectroscopic data of compounds **3** and **4** (MeOH-*d*_4_, ^1^H-NMR 600 MHz, ^13^C-NMR 150 MHz).

Position	3		4	
	*δ*_H_ (*J* in Hz)	*δ*_C_	*δ*_H_ (*J* in Hz)	*δ*_C_
1	-	211.9	-	211.2
2	-	51.2	-	52.8
3	2.80 (d, *J* = 17.0)	38.5	4.84 (s)	86.0
	3.50 (d, *J* = 17.0)			
4	7.22 (s)	126.9	7.52 (s)	127.8
5	-	146.4	-	146.2
6	-	132.5	-	140.7
7	-	136.2	-	139.1
8	-	130.1	-	131.4
9	-	153.9	-	152.7
10	4.10 (d, *J* = 9.4)	74.86	1.08 (s)	22.3
	3.48 (d, *J* = 9.4)			
11	1.14 (s)	21.8	1.28 (s)	22.8
12	2.35 (s)	19.9	2.57 (s)	22.0
13	2.74 (t, *J* = 5.5)	27.2	5.32 (dd, *J* = 4.9, 8.4)	72.7
14	3.98 (m)	65.7	3.92 (dd, *J* = 8.4, 11.5)	65.5
			3.63 (dd, *J* = 4.9,11.5)	
15	5.08 (d, *J* = 4.8)	67.5	2.74 (s)	15.2
glc-1	4.20 (d, *J* = 7.9)	104.7	4.57 (d, *J* = 7.8)	105.8
glc-2	3.21 (m)	71.5	3.36 (m)	71.7
glc-3	3.20 (m)	77.9	3.42 (m)	78.0
glc-4	3.03 (m)	74.9	3.28 (m)	75.3
glc-5	3.28 (m)	78.1	3.27 (m)	78.2
glc-6	3.78 (dd, *J* = 1.6, 11.8)	62.8	3.85 (dd, *J* = 2.0, 12.0)	62.8
	3.61 (dd, *J* = 5.7, 11.8)		3.75 (dd, *J* = 5.4, 12.0)	

## Supplementary Materials

The Supplementary Materials are available online.
