# Supplementary material for: Four New Pterosins from Pteris cretica and Their Cytotoxic Activities
Source: Molecules. 2019 Jul 30;24(15):2767. doi: 10.3390/molecules24152767 (PMC6696042; doi:10.3390/molecules24152767)

SUPPLEMENTAAL MATERIALS

Four new pterosins from *Pteris cretica* and their cytotoxic activities

Jian Lu, Caiying Peng, Shuang Cheng, Jianqun Liu, Qinge Ma and Jicheng Shu *

Key Laboratory of Modern Preparation of Traditional Chinese Medicines, Ministry of Education, Jiangxi University of Traditional Chinese Medicine, Nanchang, 330004, China;

E-Mail: lujian10120801@sina.com

*Correspondence: 20101042@jxutcm.edu.cn; Tel.: 86-791-7118658.

Received: date; Accepted: date; Published: date

Abstract: Phytochemical investigation of the aerial parts of *Pteris cretica* led to isolation and elucidation of nine pterosins, including four new pterosins, creticolacton A (**1**), 13-hydroxyl-2(*R*),3(*R*)-pterosin L (**2**), creticoside A (**3**), spelosin 3-*O*-*β*-D-glucopyranoside (**4**), together with five known pterosins (**5-9**). Their structures were identified mainly on the basis of 1D and 2D NMR spectral data, ESI-MS and literature comparisons. Compounds **1** and **3** were new type of petrosins with a six membered ring between C-14 and C-15 of positions. The new compounds were tested in *vitro* for their cytotoxic activities against four human tumor cell lines (SH-SY5Y, SGC-7901, HCT-116, Lovo). Results showed that compounds **1** and **2** exhibited cytotoxic activity against HCT-116 cells with IC_{50} value of 22.4 μM and 15.8 μM, respectively.

Keywords: *Pteris cretica* Linn.; pterosins; Cytotoxic activity.

Contents of other Supporting Information

1. UV, HRESI-MS, ^{1}H NMR, ^{13}C NMR, HSQC, HMBC, NOESY and CD spectra of 1

Figure S1 UV spectra of 1

Figure S2 HRESIMS spectra of 1

Figure S3 ^{1}H NMR spectra of 1

Figure S4 ^{13}C NMR spectra of 1

Figure S5 HSQC spectra of 1

Figure S6 HMBC spectra of 1

Figure S7 H-H NOESY spectra of 1

Figure S8 CD spectra of 1

2. UV, HRESI-MS, ^{1}H NMR, ^{13}C NMR, HSQC, HMBC, NOESY and CD spectra of 2

Figure S9 UV spectra of 2

Figure S10 HRESIMS spectra of 2

Figure S11 ^{1}H NMR spectra of 2

Figure S12 ^{13}C NMR spectra of 2

Figure S13 HSQC spectra of 2

Figure S14 HMBC spectra of 2

Figure S15 H-H NOESY spectra of 2

Figure S16 CD spectra of 2

3. UV, HRESI-MS, ^{1}H NMR, ^{13}C NMR, HSQC, HMBC, NOESY and CD spectra of 3

Figure S17 UV spectra of 3

Figure S18 HRESIMS spectra of 3

Figure S19 ^{1}H NMR spectra of 3

Figure S20 ^{13}C NMR spectra of 3

Figure S21 HSQC spectra of 3

Figure S22 HMBC spectra of 3

Figure S23 H-H NOESY spectra of 3

Figure S24 CD spectra of 3

4. UV, HRESI-MS, ^{1}H NMR, ^{13}C NMR, HSQC, HMBC, NOESY and CD spectra of 4

Figure S25UV spectra of 4

Figure S26 HRESIMS spectra of 4

Figure S27 ^{1}H NMR spectra of 4

Figure S28 ^{13}C NMR spectra of 4

Figure S29 HSQC spectra of 4

Figure S30 HMBC spectra of 4

Figure S31 H-H NOESY spectra of 4

Figure S32 CD spectra of 4

1. UV, HRESI-MS, ^{1}H NMR, ^{13}C NMR, HSQC, HMBC, NOESY and CD spectra of 1

Figure S1 UV spectra of 1

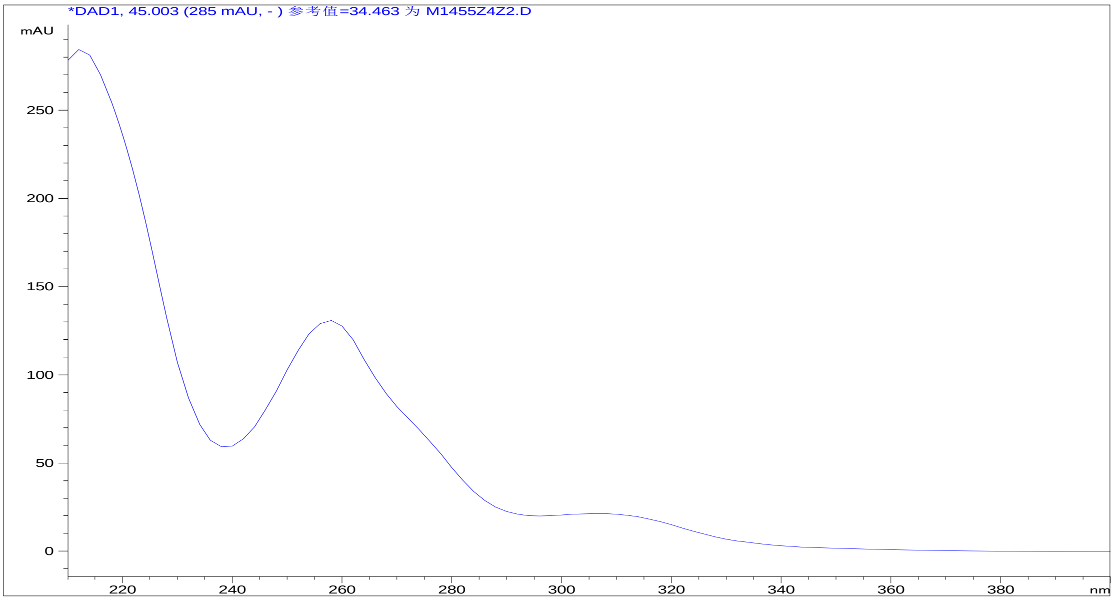

Figure S2 HRESIMS spectra of 1

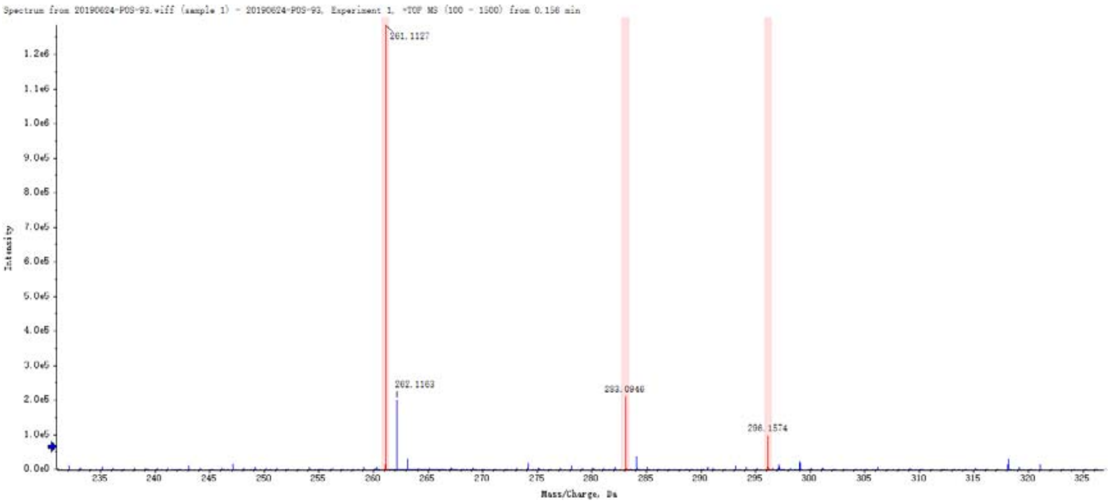

Figure S3 ^{1}H NMR spectra of 1

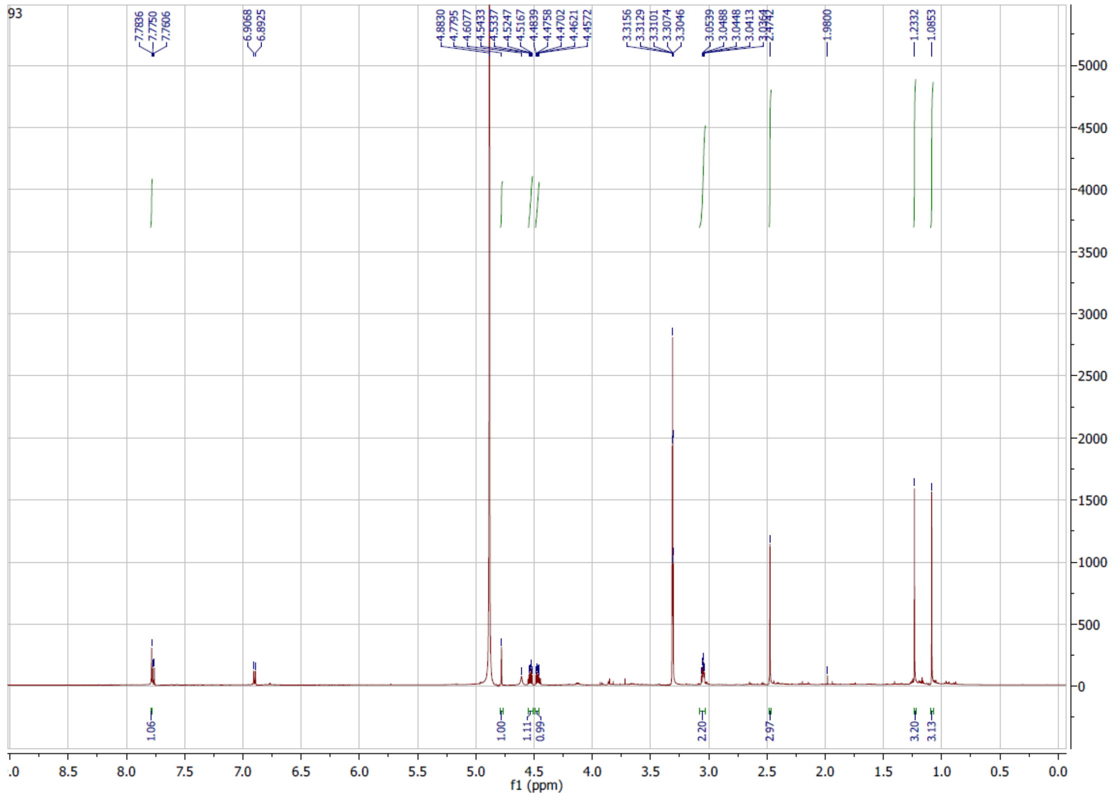

Figure S4 ^{13}C NMR spectra of 1

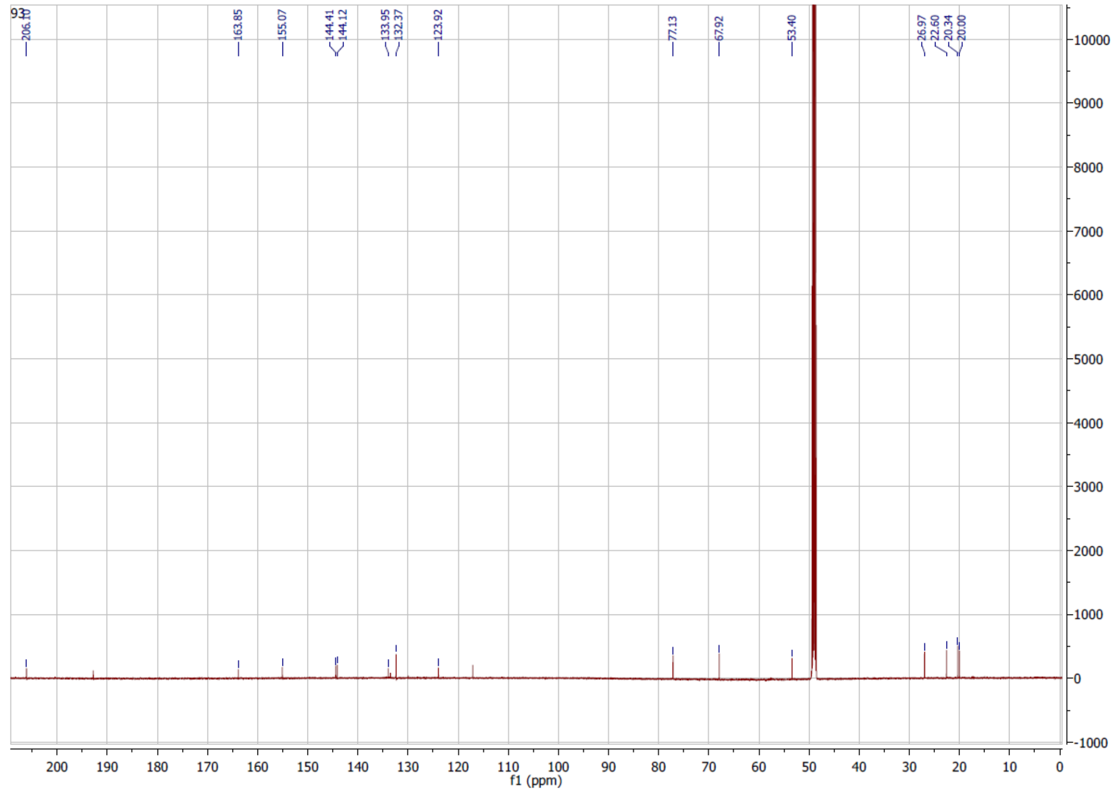

Figure S5 HSQC spectra of 1

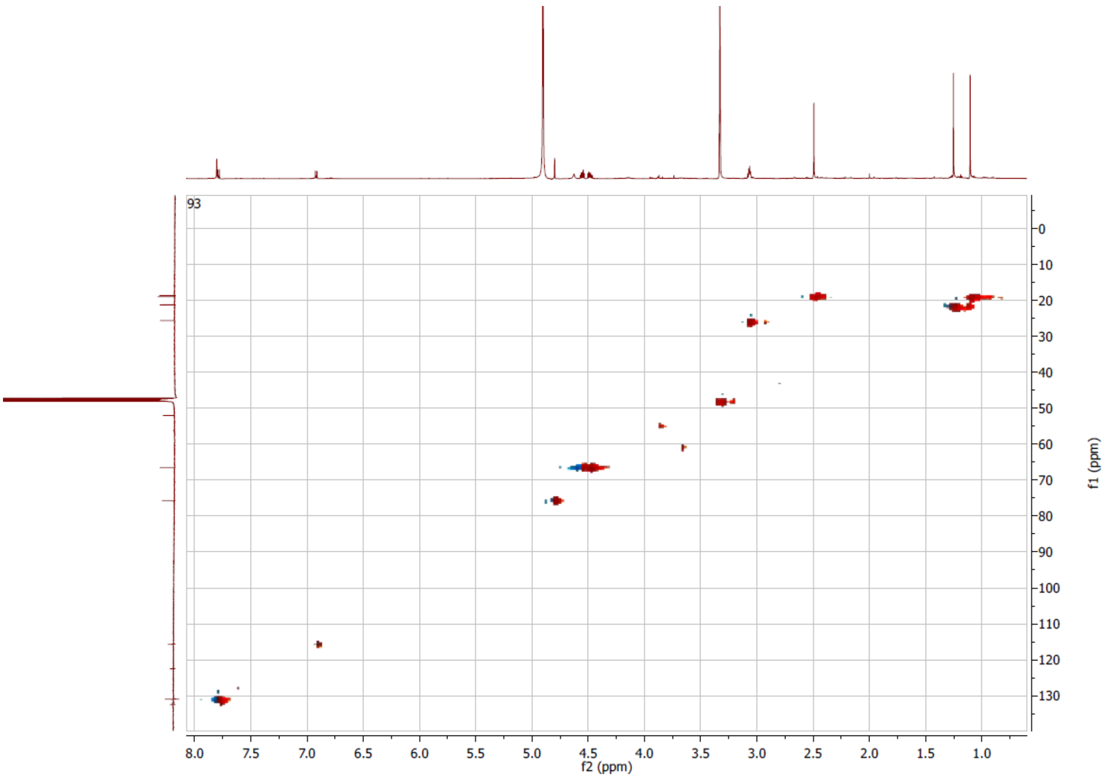

Figure S6 HMBC spectra of 1

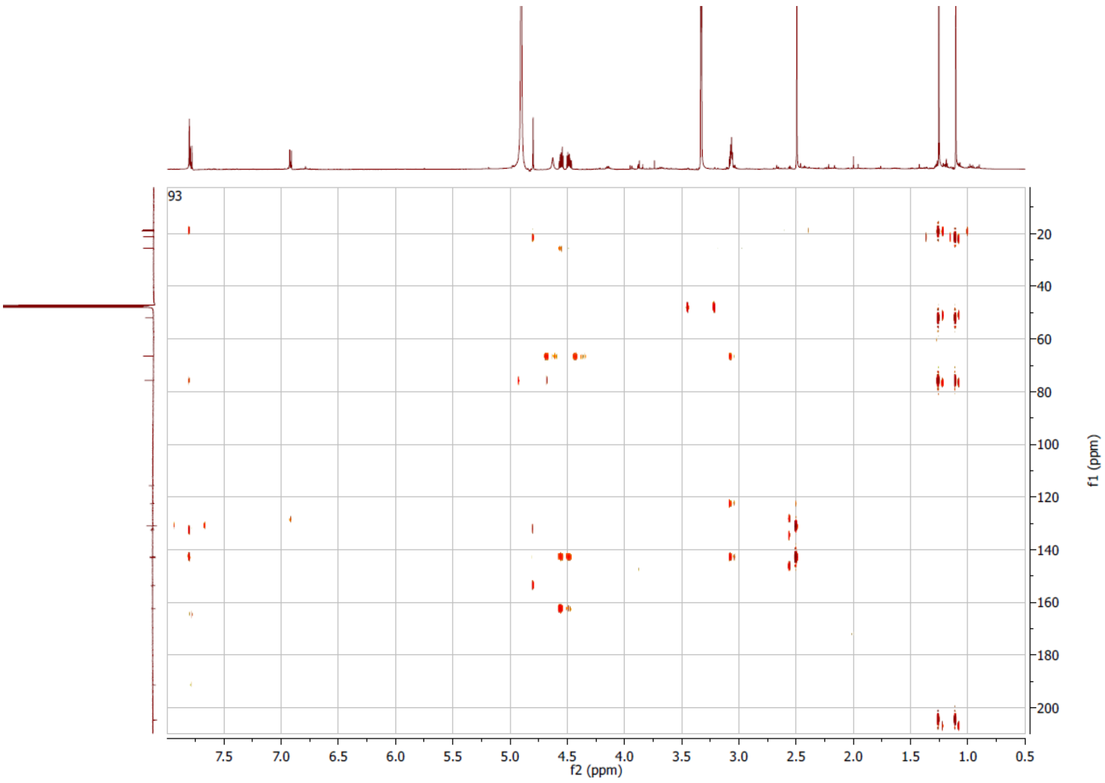

Figure S7 H-H NOESY spectra of 1

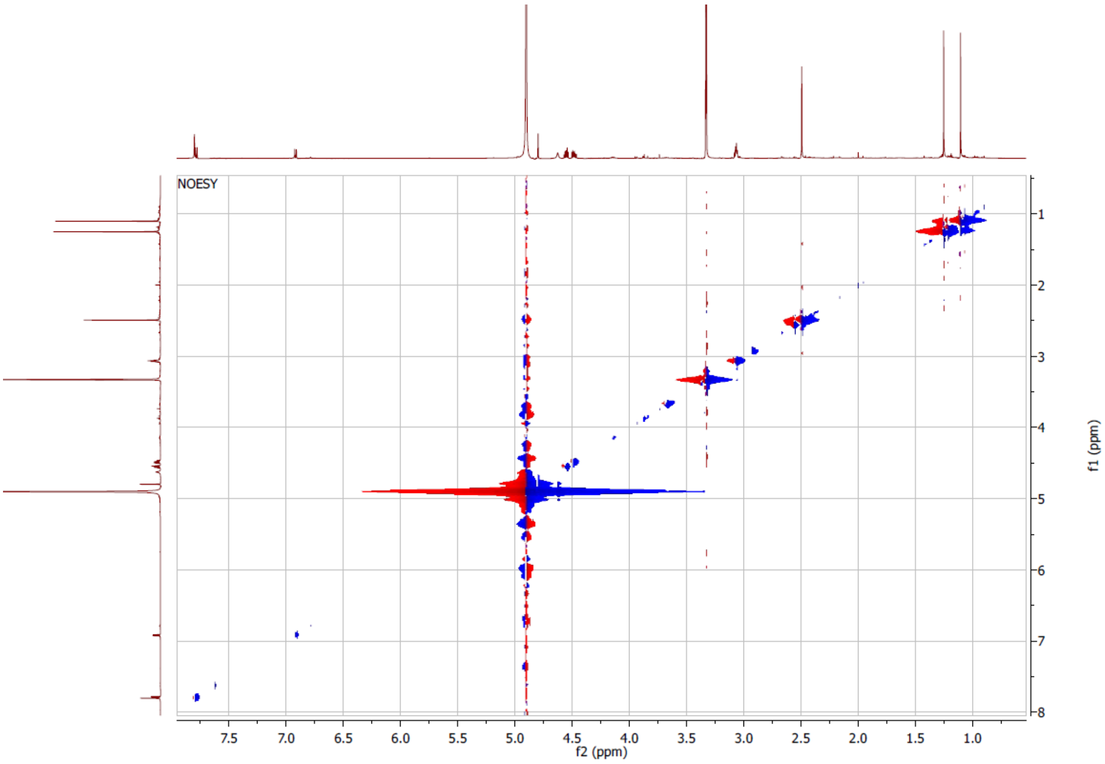

Figure S8 CD spectra of 1

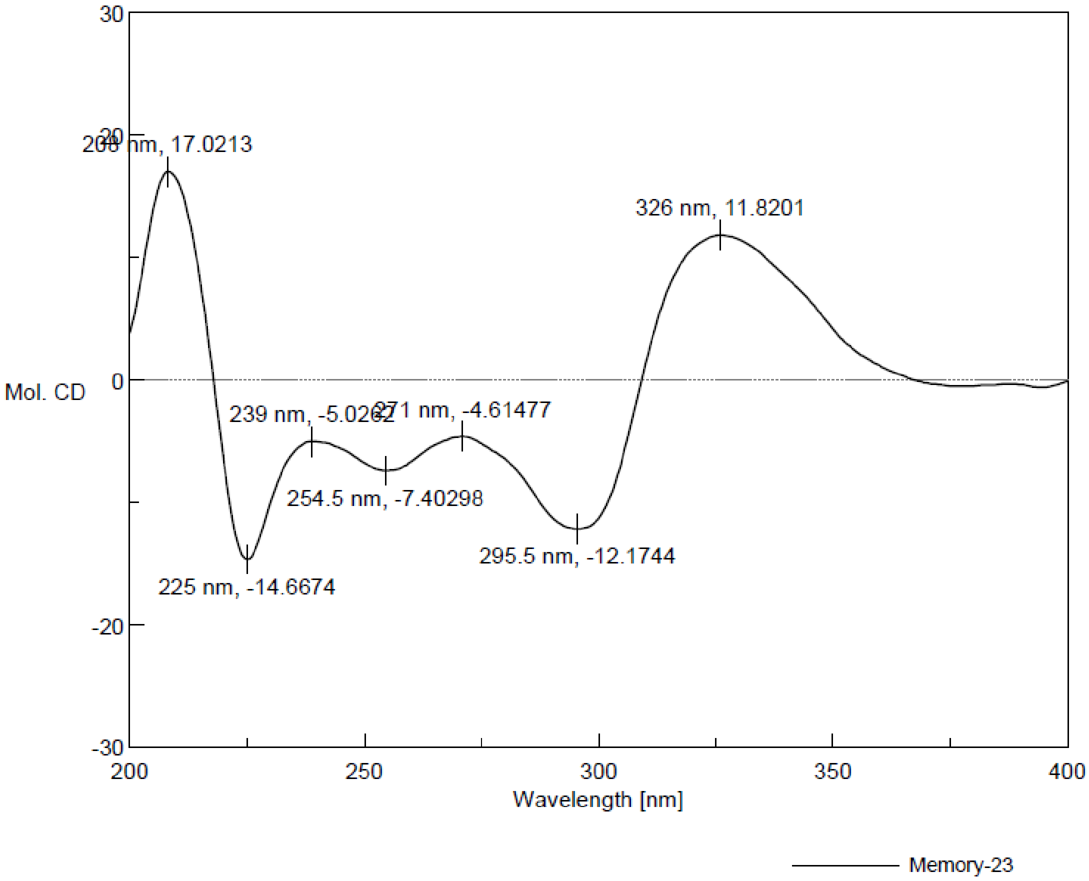

2. UV, HRESI-MS, ^{1}H NMR, ^{13}C NMR, HSQC, HMBC, NOESY and CD spectra of 2

Figure S9 UV spectra of 2

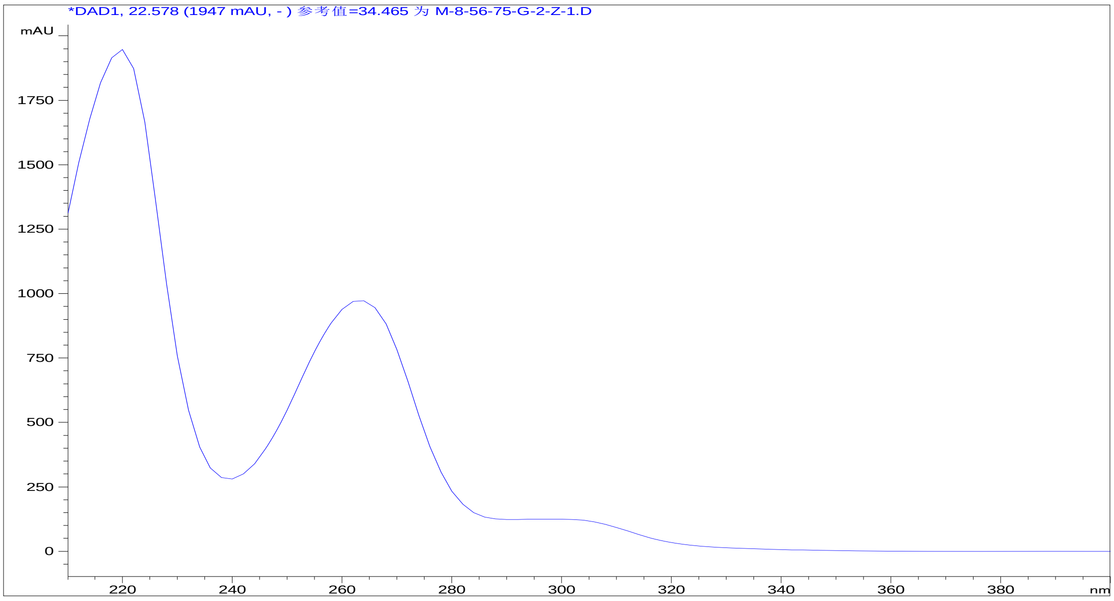

Figure S10 HRESIMS spectra of 2

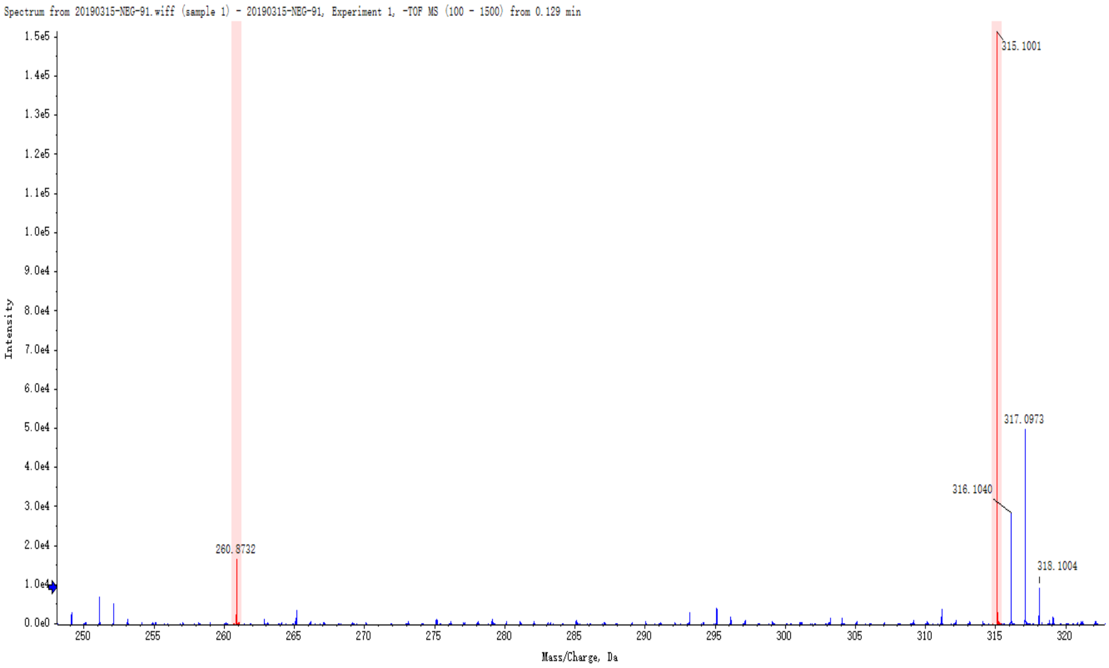

Figure S11 ^{1}H NMR spectra of 2

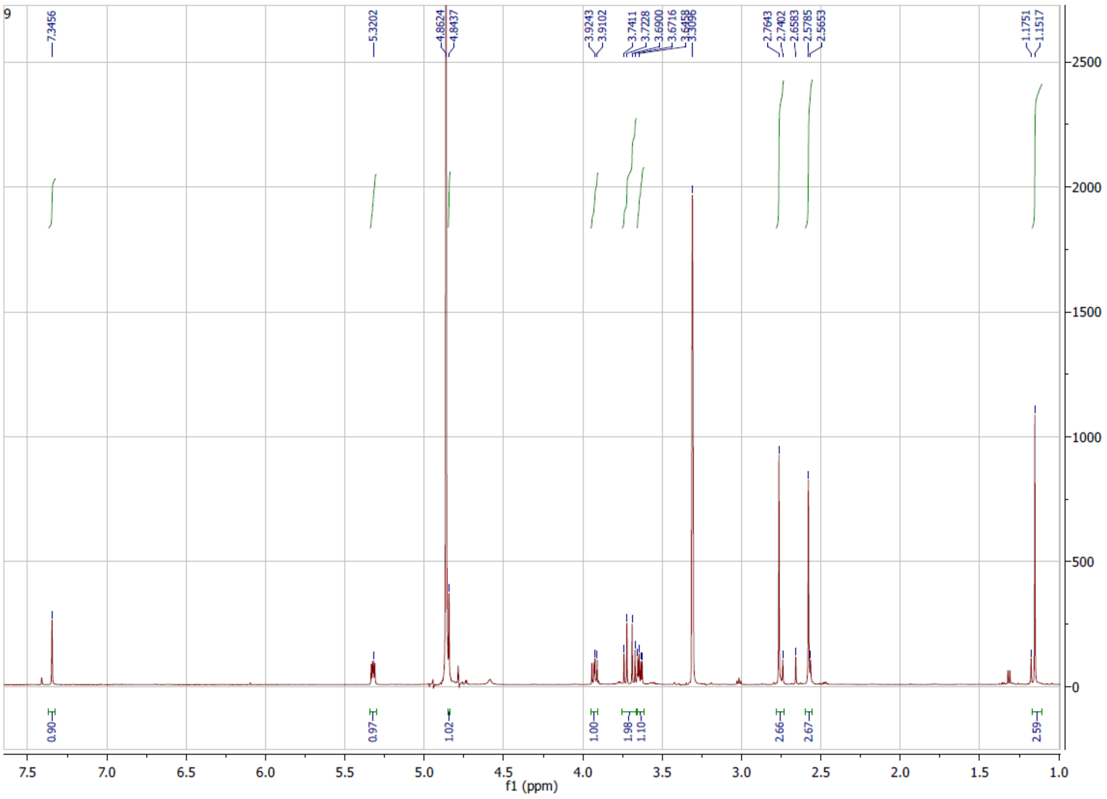

Figure S12 ^{13}C NMR spectra of 2

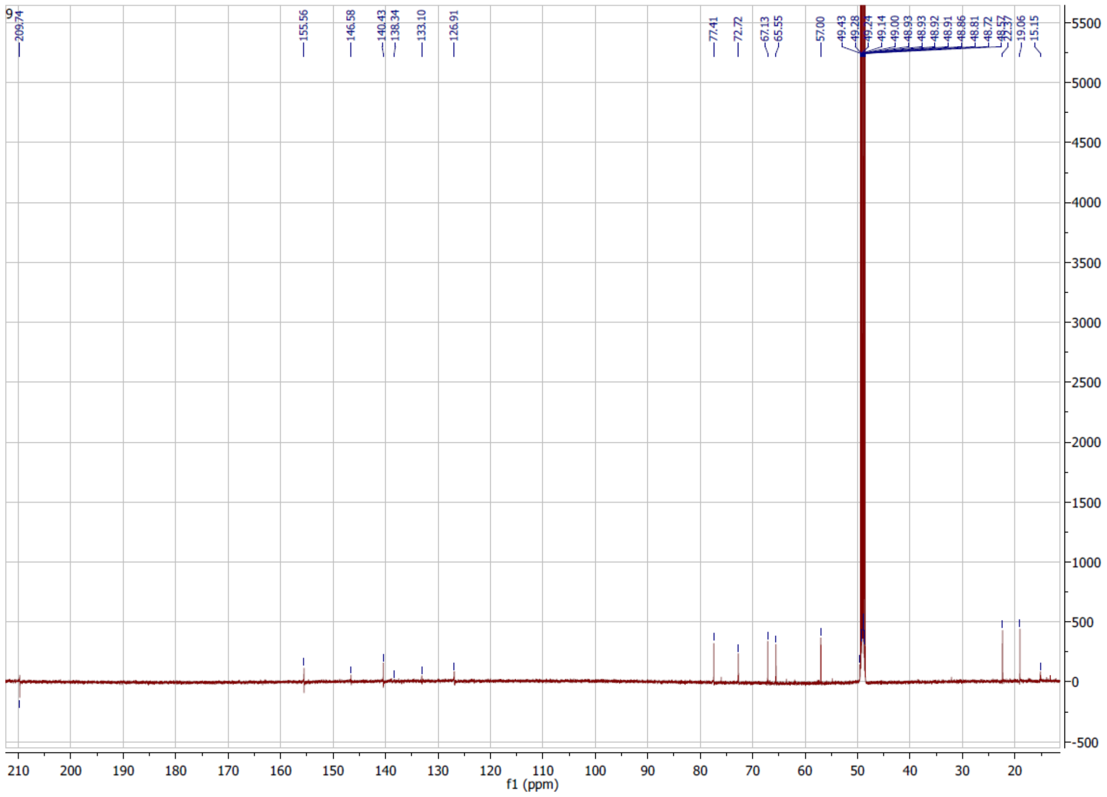

Figure S13 HSQC spectra of 2

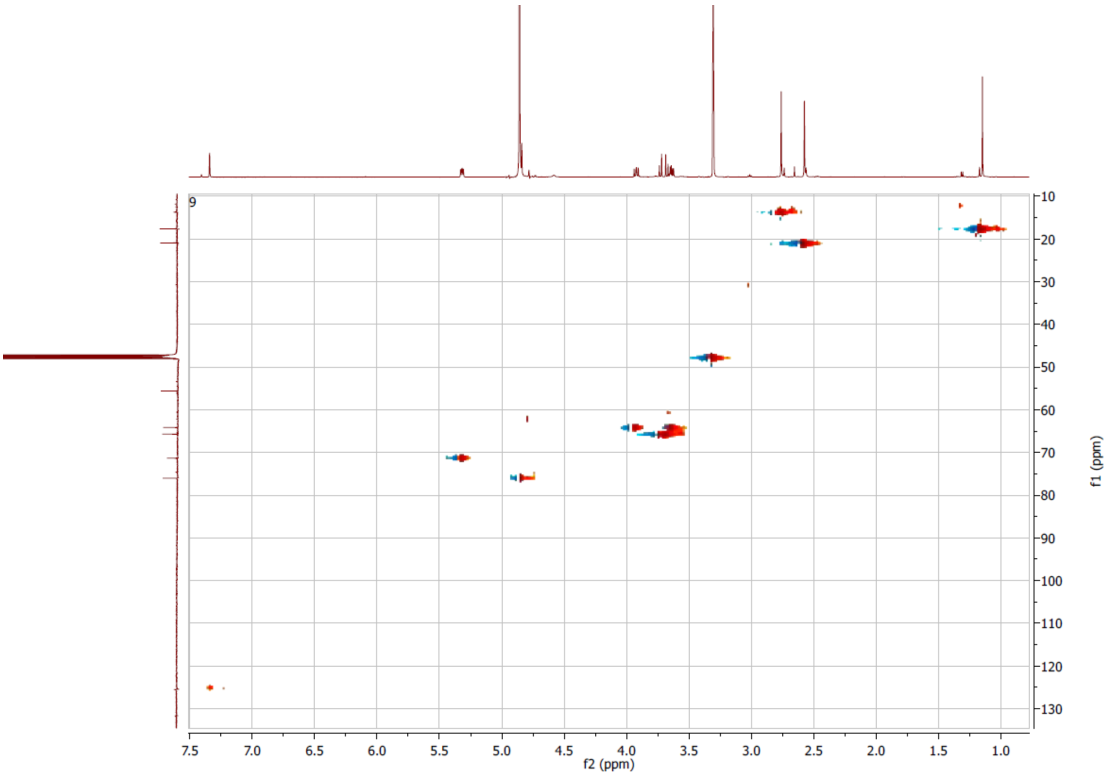

Figure S14 HMBC spectra of 2

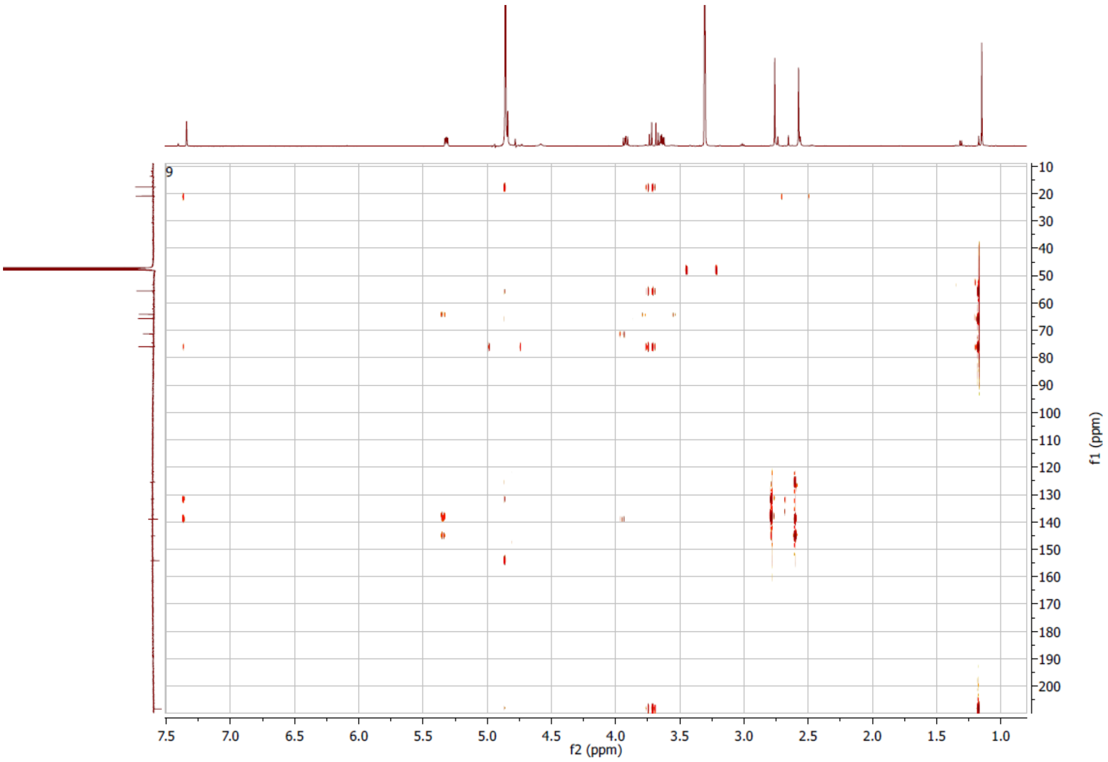

Figure S15 H-H NOESY spectra of 2

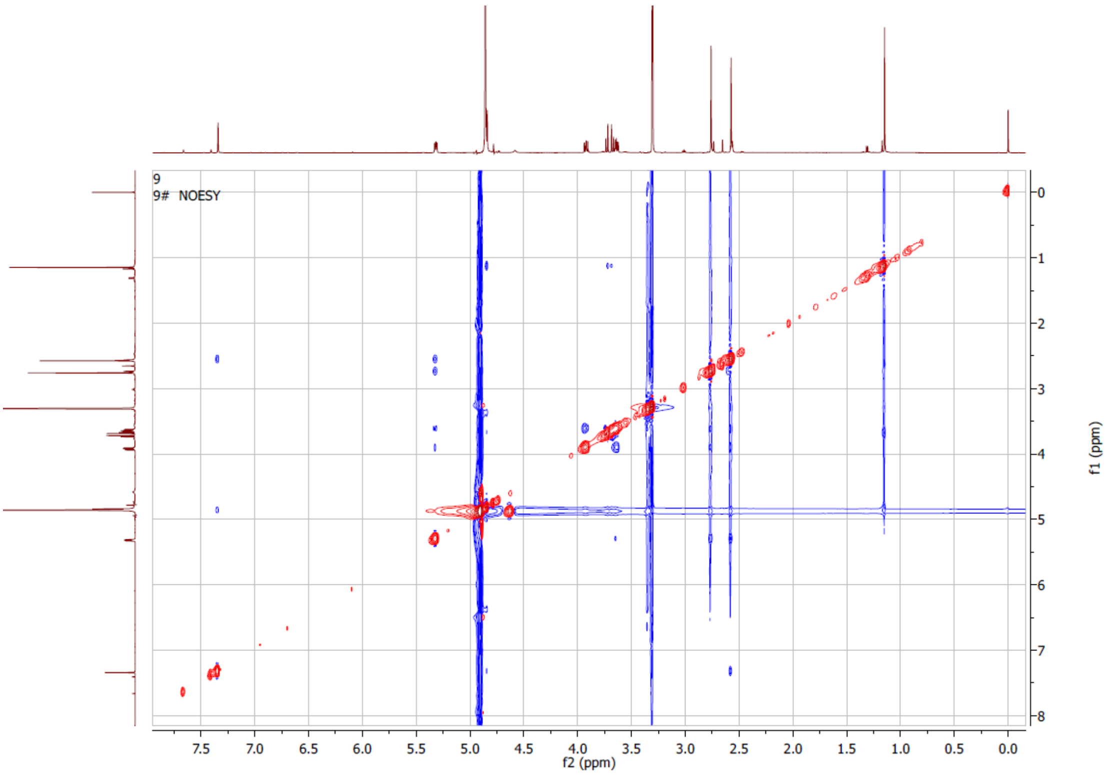

Figure S16 CD spectra of 2

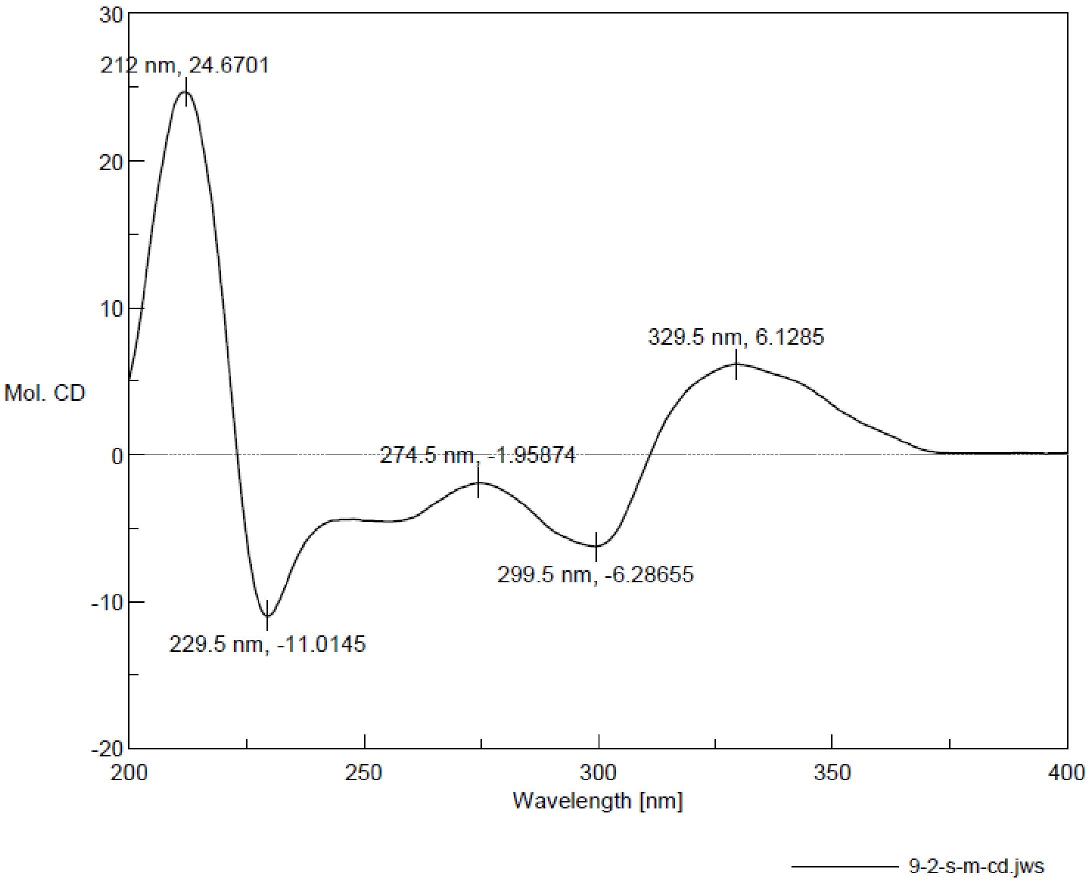

3. UV, HRESI-MS, ^{1}H NMR, ^{13}C NMR, HSQC, HMBC, NOESY and CD spectra of 3

Figure S17 UV spectra of 3

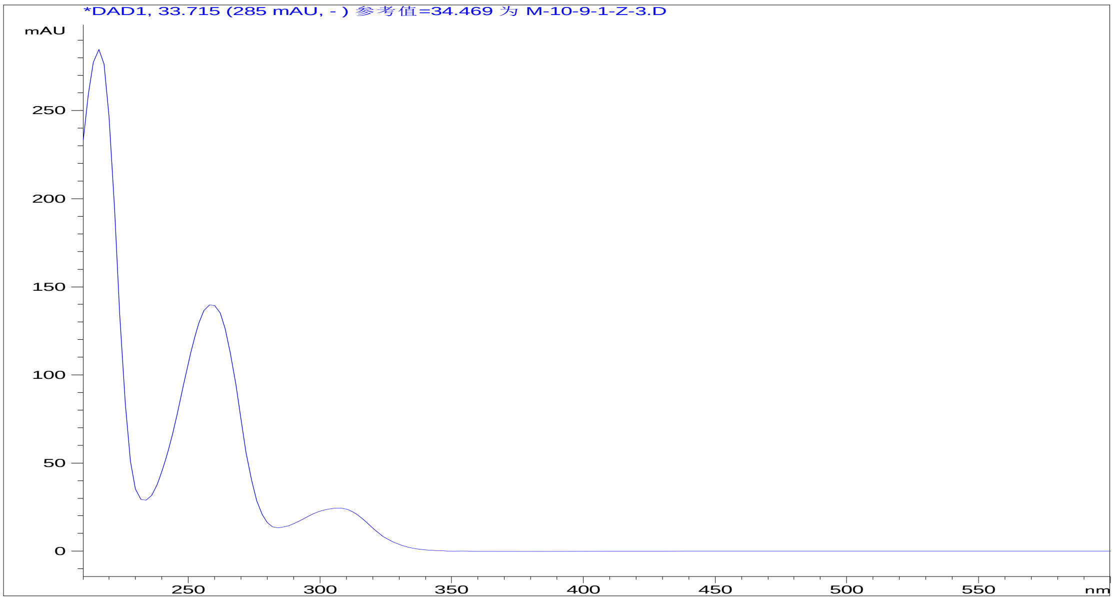

Figure S18 HRESIMS spectra of 3

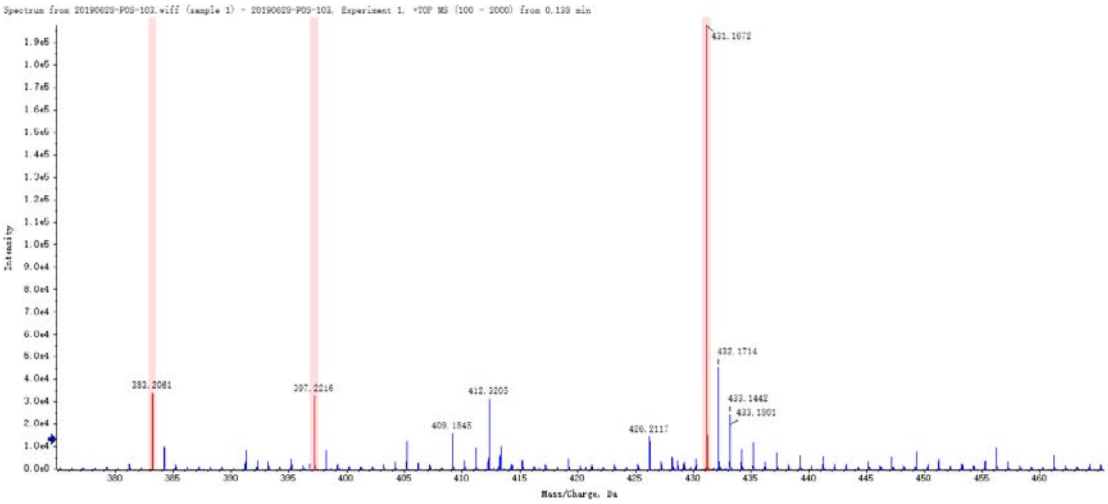

Figure S19 ^{1}H NMR spectra of 3

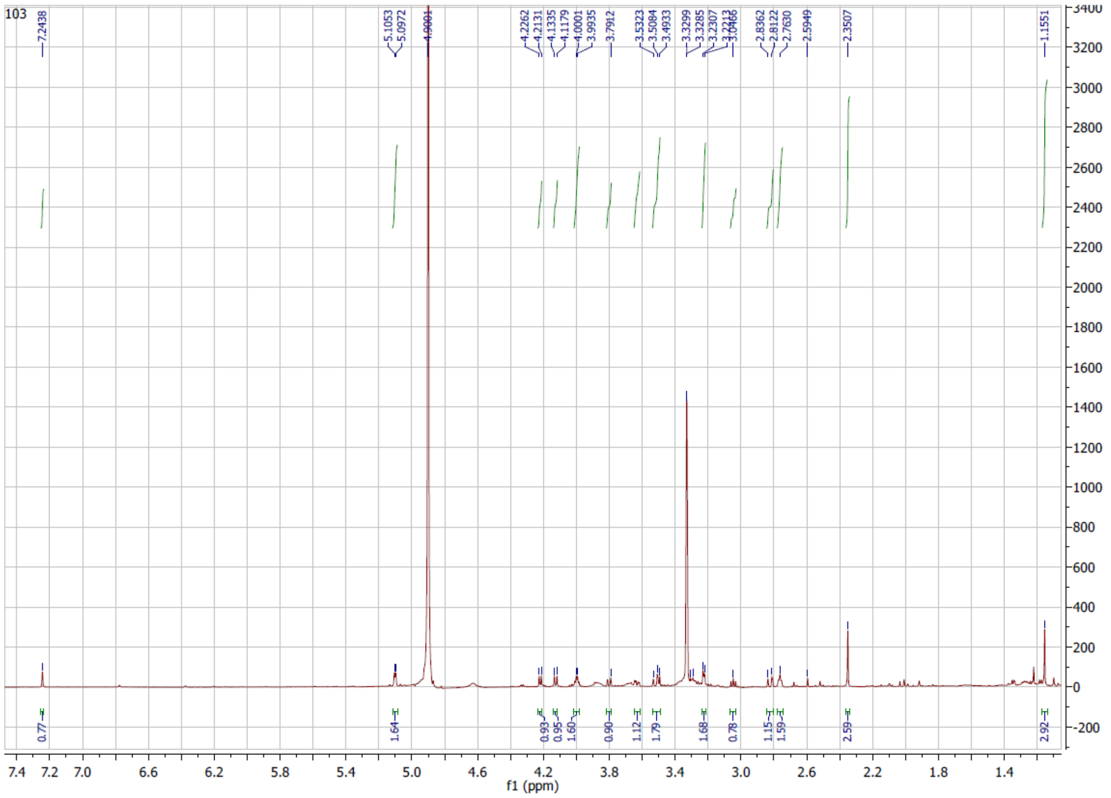

Figure S20 ^{13}C NMR spectra of 3

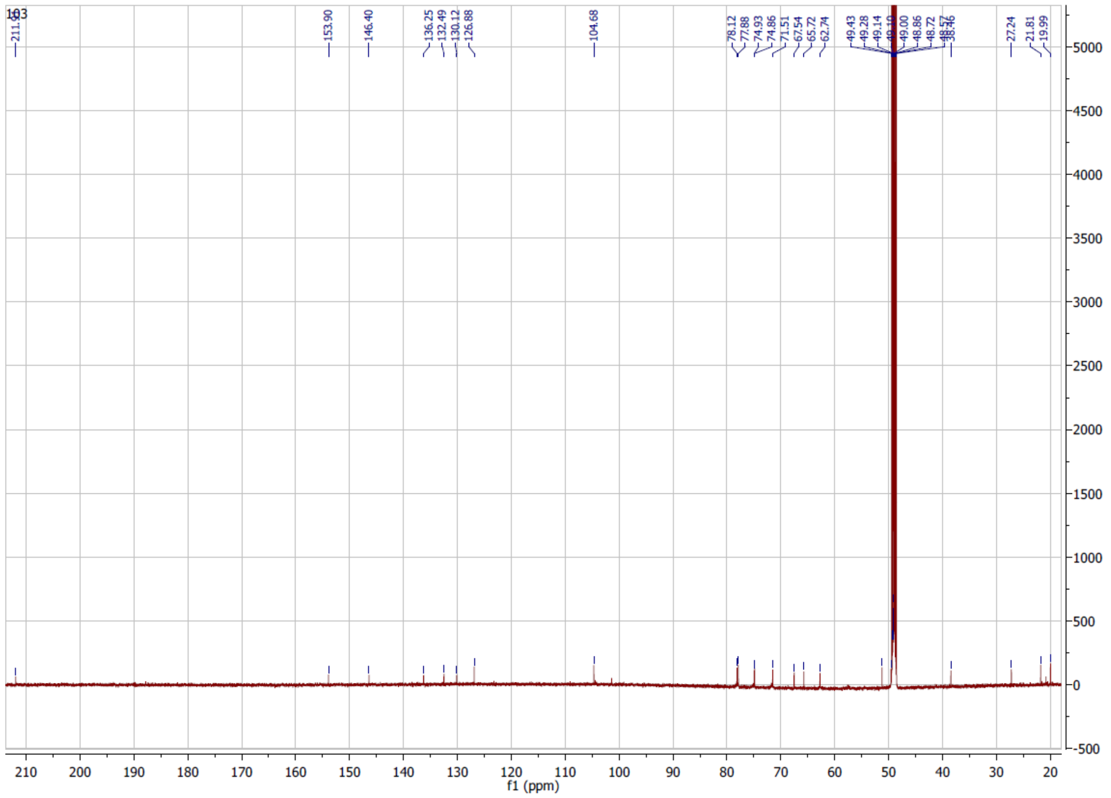

Figure S21 HSQC spectra of 3

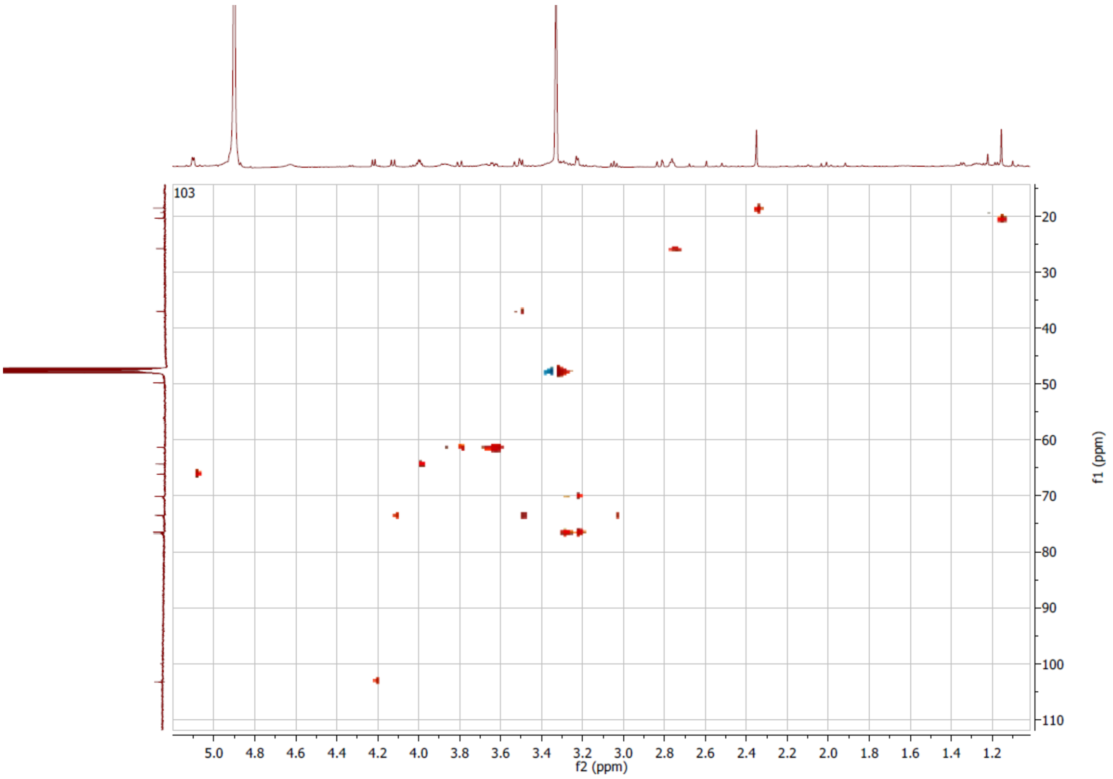

Figure S22 HMBC spectra of 3

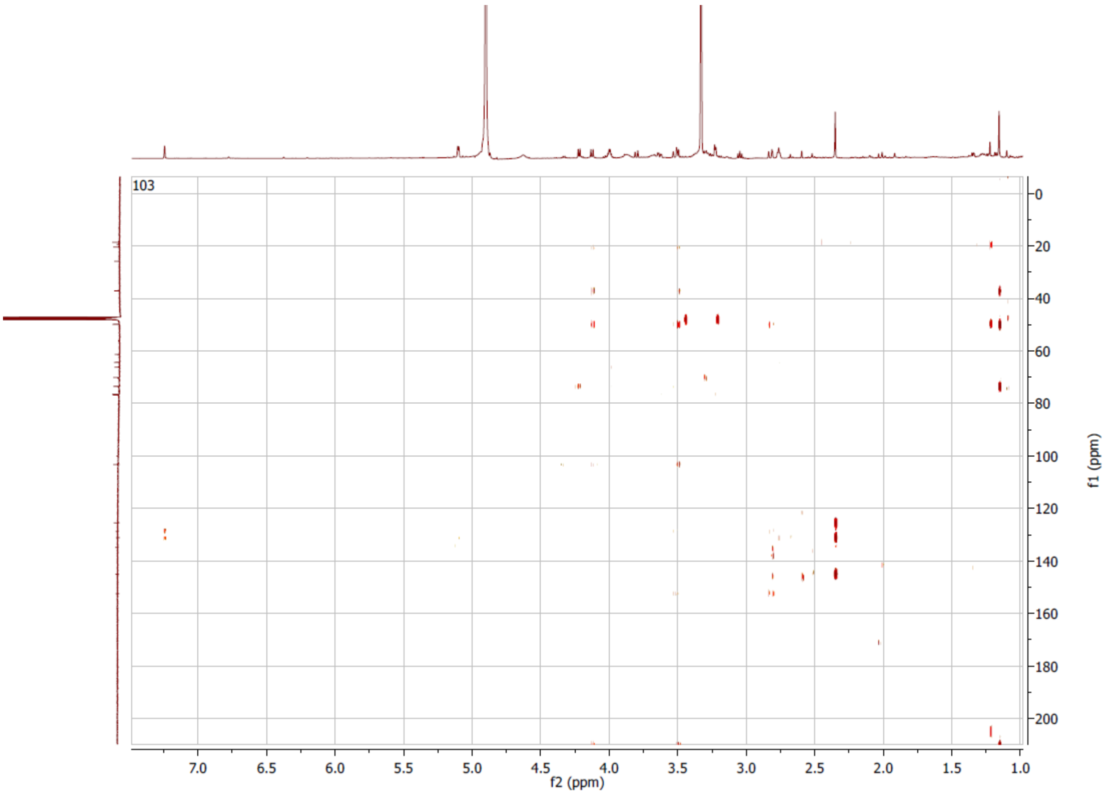

Figure S23 H-H NOESY spectra of 3

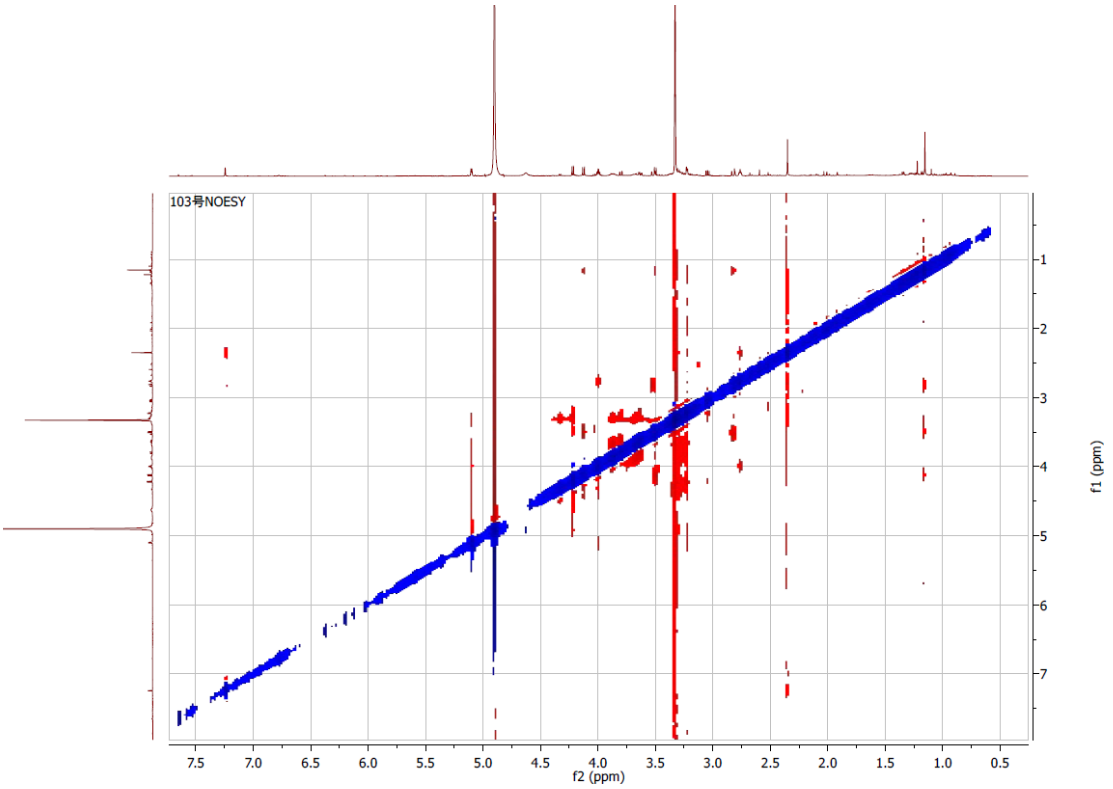

Figure S24 CD spectra of 3

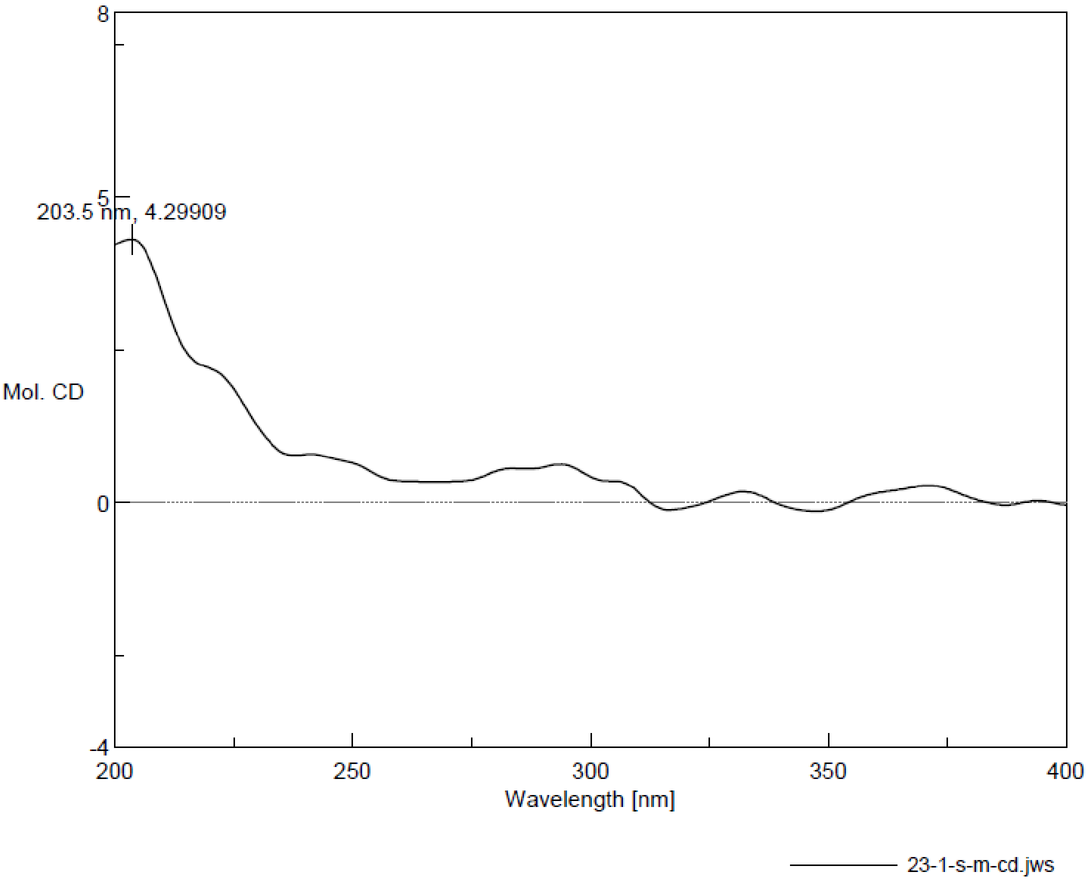

4. UV, HRESI-MS, ^{1}H NMR, ^{13}C NMR, HSQC, HMBC, NOESY and CD spectra of 4

Figure S25UV spectra of 4

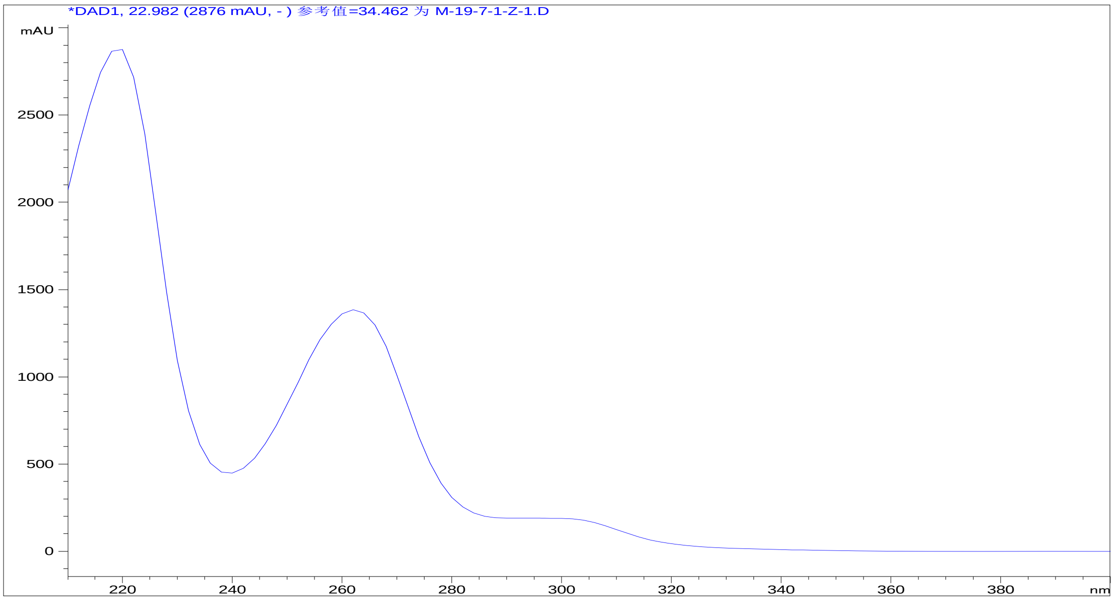

Figure S26 HRESIMS spectra of 4

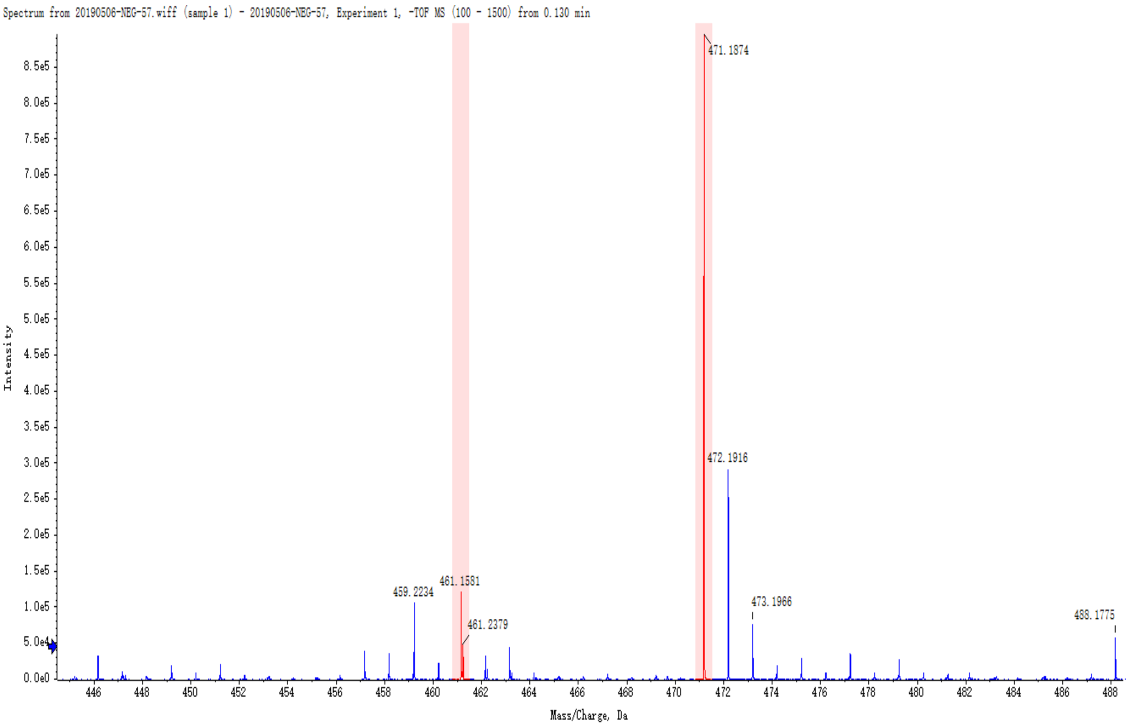

Figure S27 ¹H NMR spectra of 4

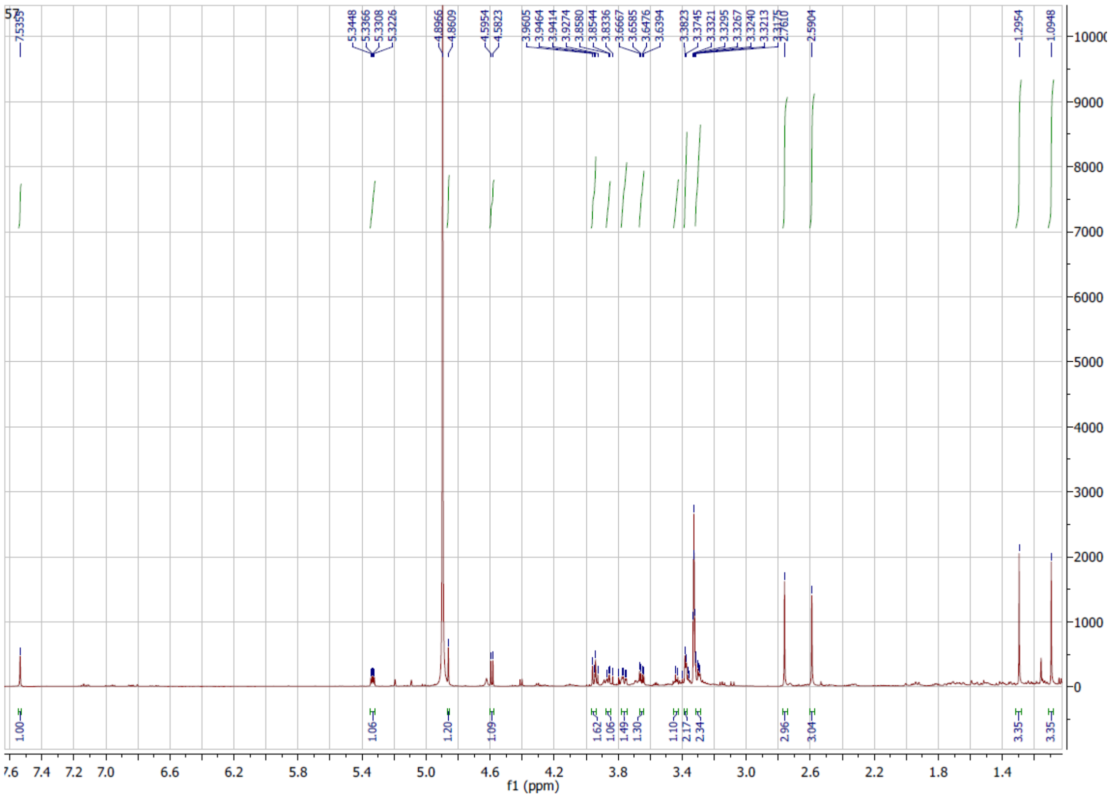

Figure S28 ¹³C NMR spectra of 4

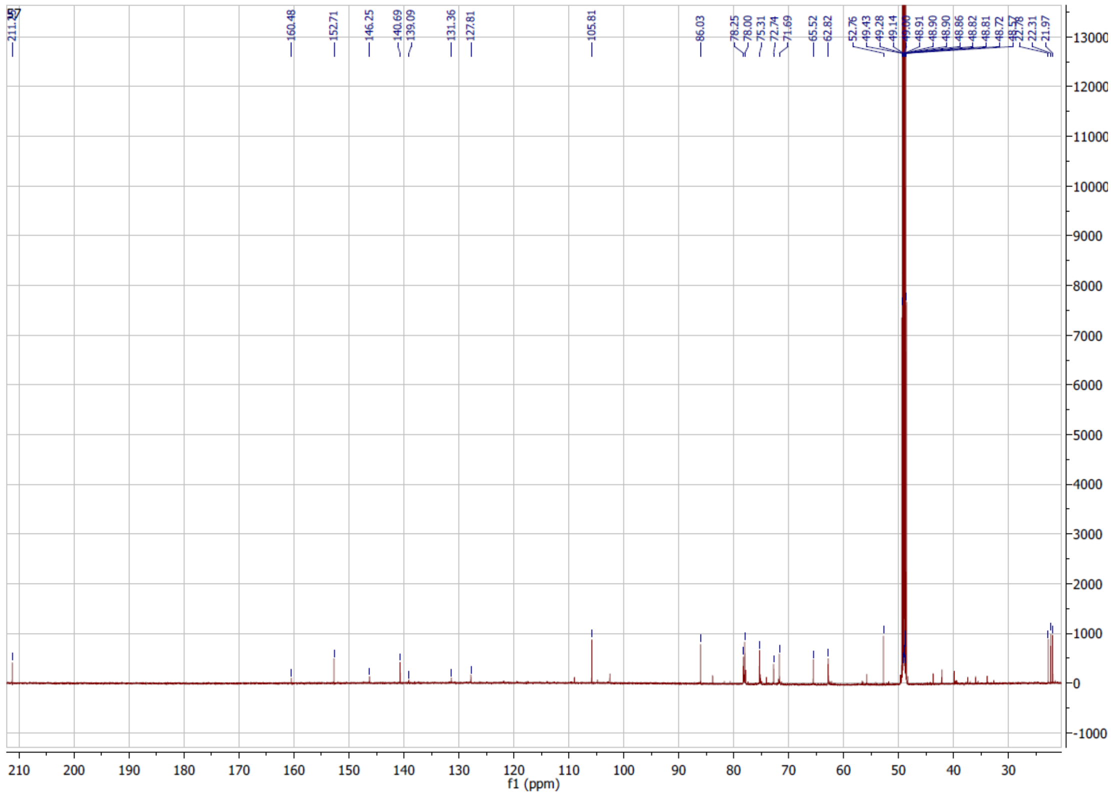

Figure S29 HSQC spectra of 4

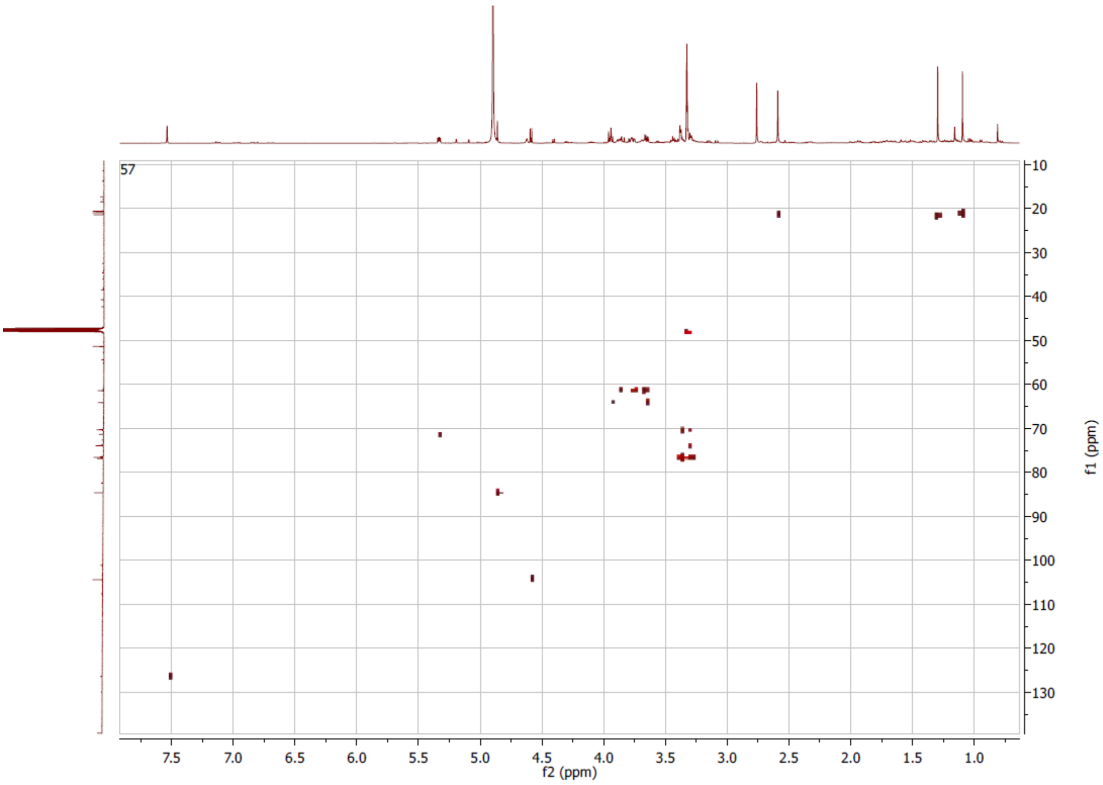

Figure S30 HMBC spectra of 4

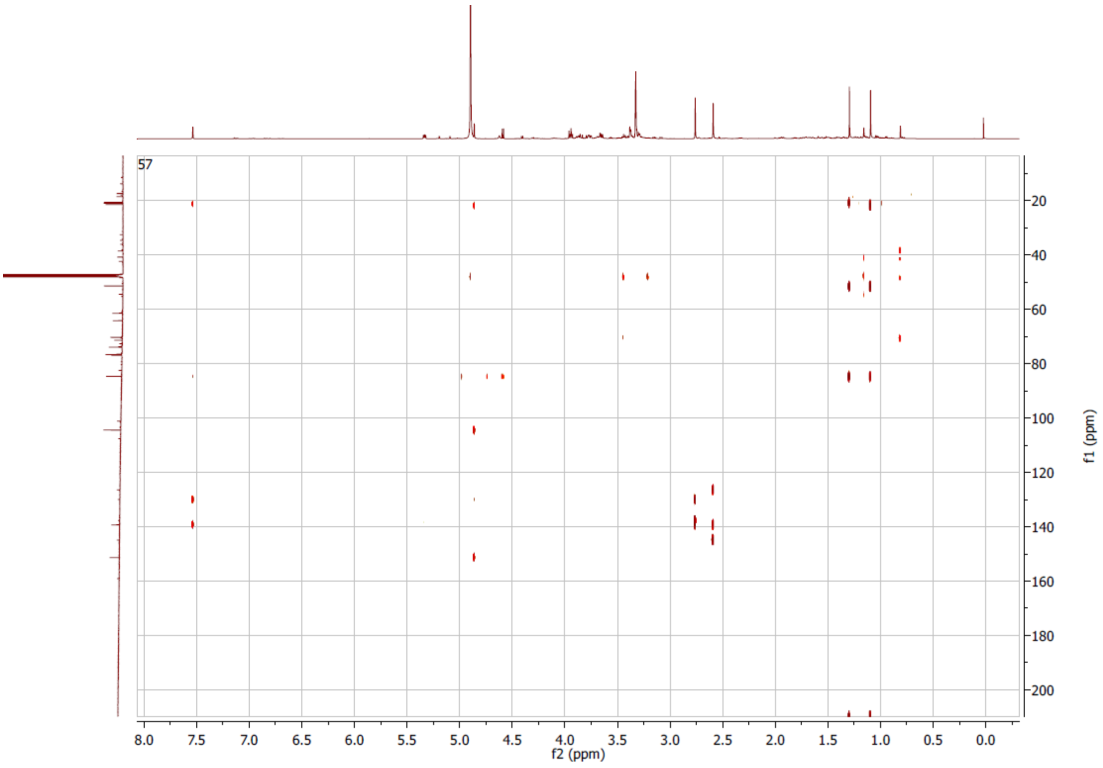

Figure S31 H-H NOESY spectra of 4

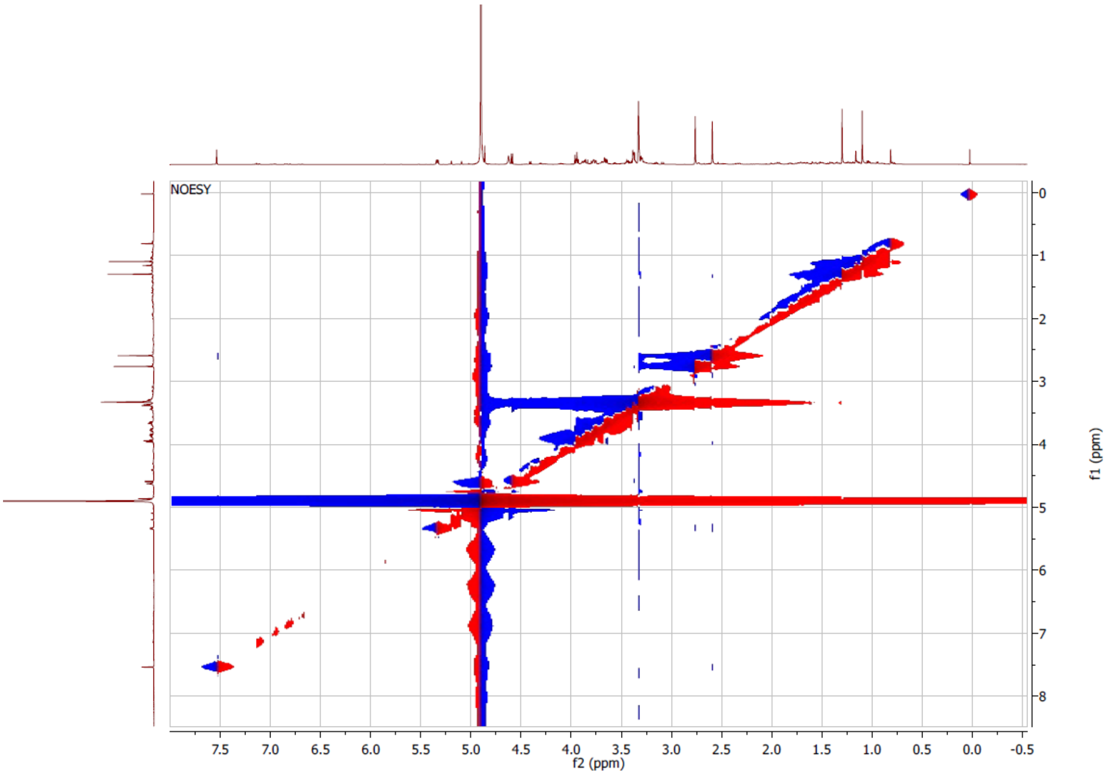

Figure S32 CD spectra of 4

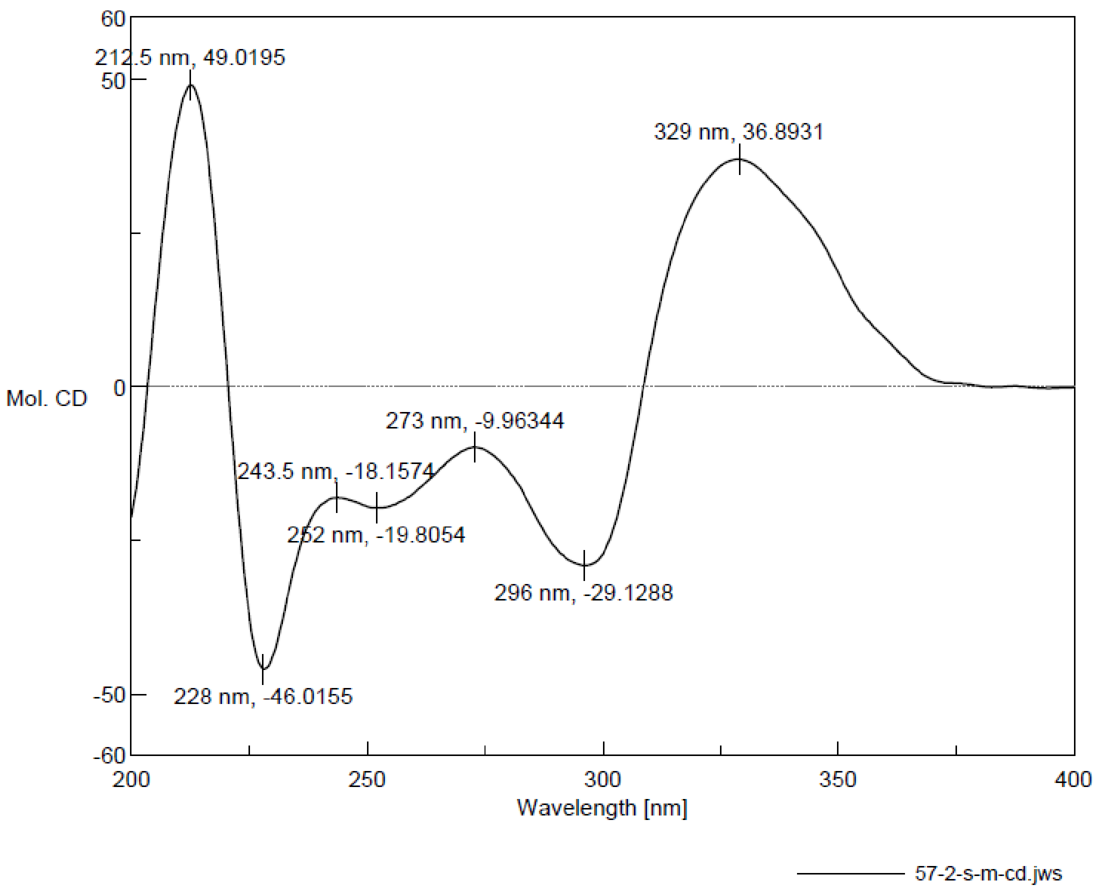

Supplement: Supplementary file 1 [file molecules-24-02767-s001.pdf]
